# Supplementary material for: Effectiveness and Coverage of Treatment for Severe Acute Malnutrition Delivered by Community Health Workers in the Guidimakha Region, Mauritania
Source: Children (Basel). 2021 Dec 4;8(12):1132. doi: 10.3390/children8121132 (PMC8700149; doi:10.3390/children8121132)
Supplement: Supplementary file 1 [file children-08-01132-s001.zip › Suplemmentary material 3.pdf]

MODULE 1.

ACCUEIL, SIGNES DE DANGER ET RÉFÉRENCEMENT

Module de Formation des Agents de Santé Communautaire sur le Protocole PCIMA

ACTION CONTRE LA FAIM

OBJECTIFS PÉDAGOGIQUES

A la fin de ce module, l'agent de santé communautaire (ASC) doit être capable de:

1. Disposer des mesures minimales pour accueillir les enfants et les accompagnantes
2. Référer les enfants présentant des complications médicales vers les structures de santé

CE DONT L'ASC A BESOIN

avoir un espace pour que la mère puisse s'asseoir **(Hangar)**

la disponibilité d'un dispositif de lavage des mains au savon

IDENTIFIER LE PROBLÈME DE L'ENFANT

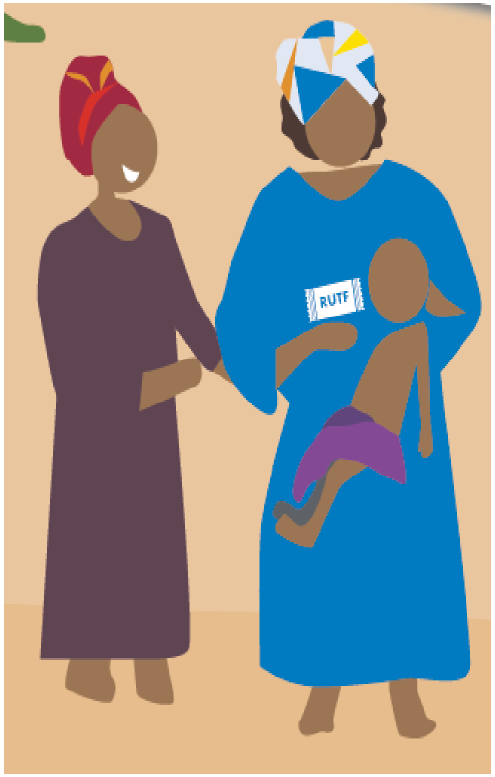

Demandez à la mère :

1. De quoi l'enfant se plaint-il ?
2. Combien de jours l'enfant a-t-il été malade ?
3. A-t-il été traité ou emmené dans un autre lieu pour y être soigné ?

Identifier le problème de l'enfant

EXAMEN PHYSIQUE DE L'ENFANT

1. prendre la température corporelle

2. Après avoir touché l'enfant
3. Après avoir salué la mère ou l'accompagnant
4. Avant d'effectuer toute intervention de nettoyage ou d'aseptique,
5. Après exposition aux fluides corporels (urines, sang)

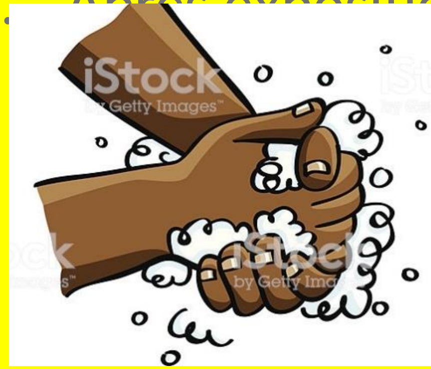

RECHERCHE DE SIGNES DE DANGER DE L'ENFANT

Interrogatoire. Ecouter à la mère	
Diarrhée de plus de 2 semaines	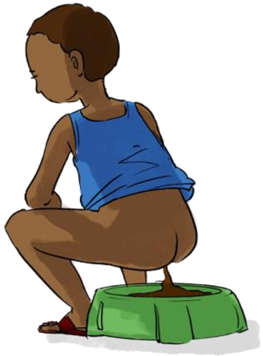
Diarrhée avec du sang	
Toux et fièvre de plus de 2 semaines (14 jours)	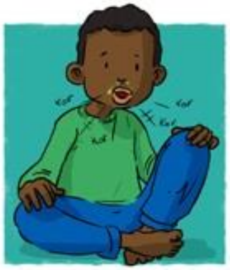
Fièvre de plus de 7 jours	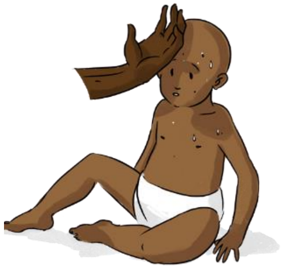

RECHERCHE DE SIGNES DE DANGER DE L'ENFANT

Observation de l'enfant	
Incapable de boire ou de téter	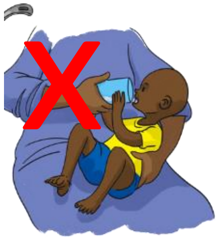 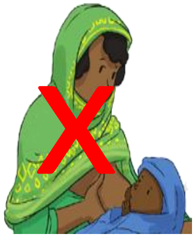
Vomit tout ce qu'il consomme	
Sommeil inhabituel ou inconscient	
Convulsion	

RECHERCHE DE SIGNES DE DANGER DE L’ENFANT

Examen physique de l’enfant	
Prendre le temperature	
Pâleur palmaire	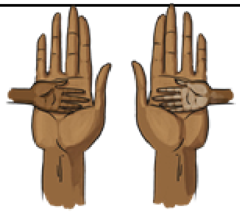
Respiration difficile et rapide	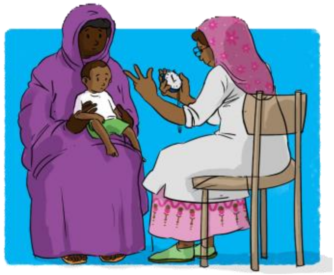
Raideur de la nuque	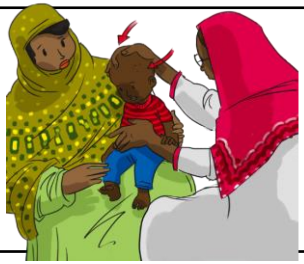
Périmètre brachial rouge + test de l’appétit médiocre + Œdème +++	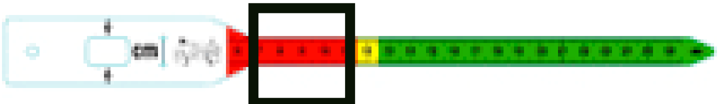

RECHERCHE DE SIGNES DE DANGER DE L'ENFANT

Examen physique de l'enfant	
Pâleur palmaire	Comparer les paumes de l'ASC à celle de l'enfant : la pâleur montre une main blanche tandis qu'une bonne coloration montrera une rougeur de la paume de la main de l'enfant
Respiration difficile et rapide	Compter les respirations par minute, rechercher un tirage sous-costal, écouter une respiration sifflante,
Raideur de la nuque	Regarder si l'enfant remue et fléchit le cou sans difficulté pendant qu'il regarde autour de lui. Si l'enfant remue et plie le cou, il n'a pas de raideur.<br>Pour rechercher la raideur du cou, demander à la mère de tenir l'enfant assis sur ses cuisses ; l'ASC place une main derrière la tête de l'enfant au niveau du cou et fléchit doucement en posant l'autre main sur la poitrine de l'enfant. Si le cou se plie aisément et le « menton touche la poitrine » l'enfant n'a pas de raideur du cou ou raideur de la nuque. Si le menton ne touche pas la

RECHERCHE DE SIGNES DE DANGER DE L'ENFANT

Examen physique de l'enfant	
Périmètre brachial rouge	PB rouge avec des autres signes de danger
Œdème +++	

PRENDRE LE PÉRIMÈTRE BRACHIAL (PB)

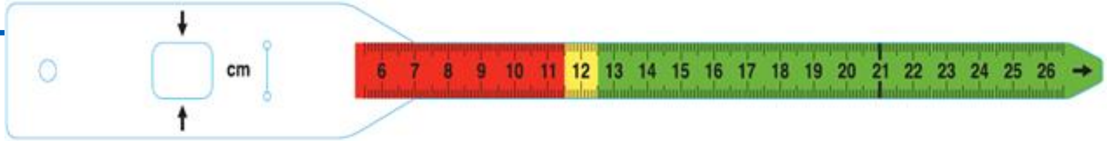

Trouvez, sur le bras de l'enfant, le point central

Apposez l'extrémité la plus large de la bandelette sur ce point central.

Enroulez l'autre extrémité de la bandelette autour du bras de l'enfant

Pas trop séré

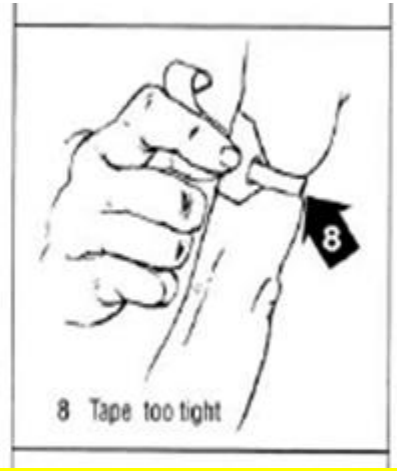

Pas trop lâche

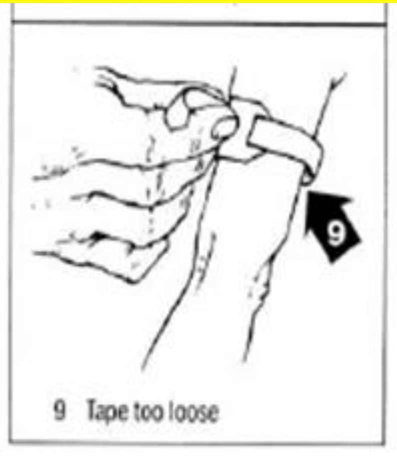

VÉRIFIER LES ŒDÈMES BILATÉRAUX

1. On exerce une pression normale avec le pouce sur les deux pieds pendant au moins trois secondes
2. Si l'empreinte du pouce persiste sur les deux pieds

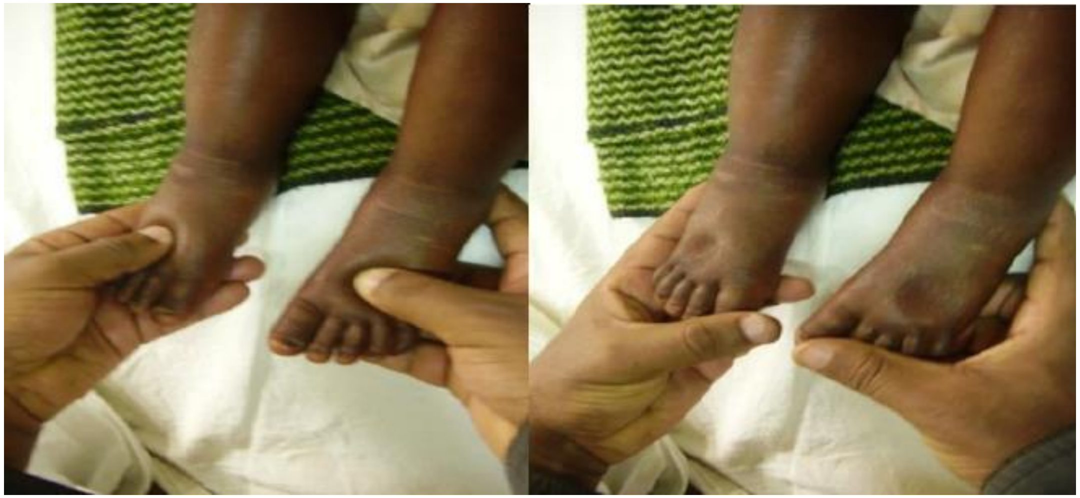

ENFANT MALNUTRITION AIGUE SEVERE

Sévérité des oedèmes Codification

- **Œdèmes Sévères+++** : généralisés soit incluant les 2 pieds, jambes, mains, bras et le visage

TRANSFÉRER CENTRE DE SANTÉ

1. Ecouter à la mère

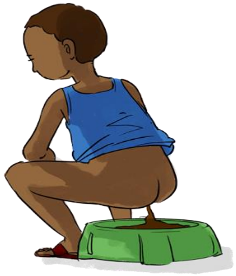
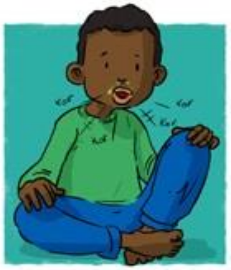
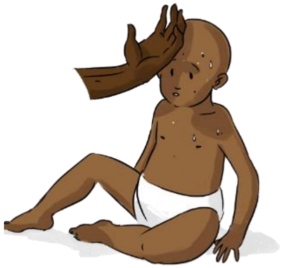

2. Observation de l'enfant

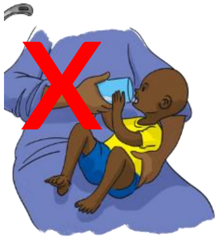
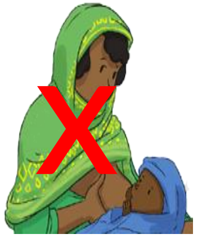

2. Examen physique de l'enfant

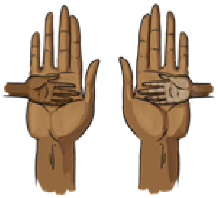
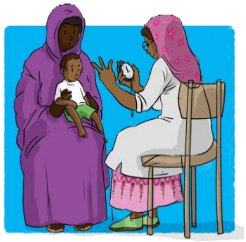
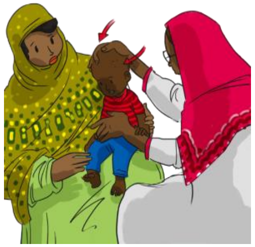
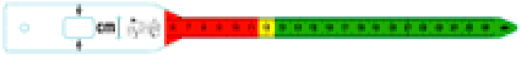

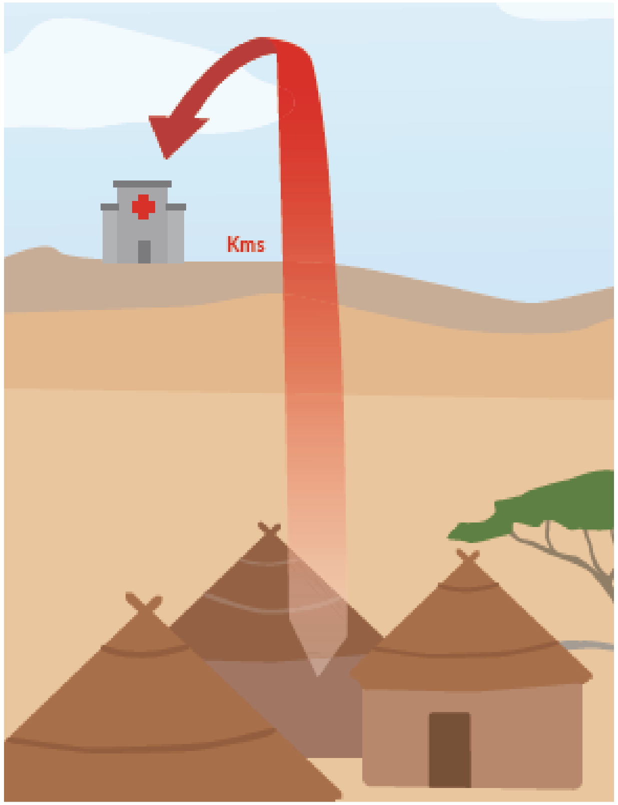

AVANT DE RÉFÉRER LES SIGNES IDENTIFIÉS

Continuer à faire téter ou boir sen cours de route si l'enfant peut

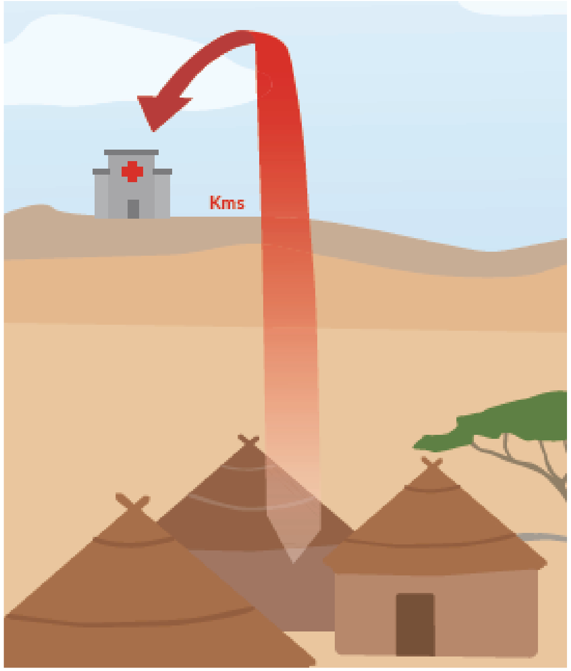

Diarrhée
Donner du SRO,

Diarrhée + PB rouge
Donner de l'eau sucrée

Fièvre+ TDR positive
Donner 1 dose artesunate

Fièvre+ infection respiratoires
Donner 1 dose amoxicilline

MESSAGES CLÉS

Question	réponse
1.Quelles sont les moments que l'ASC doit se laver les mains ?	• Prendre la température corporelle<br>• Après avoir touché l'enfant<br>• Après avoir salué la mère ou l'accompagnant<br>• Avant d'effectuer toute intervention de nettoyage ou d'aseptique,<br>• Après exposition aux fluides corporels (urines, sang)
2. Trois premières choses que l'ASC doit toujours faire pour la recherche des signes de danger	Ecouter la mère ou la gardienne de l'enfant<br>Observer l'enfant<br>Examen physique de l'enfant

MESSAGES CLÉS

Question	réponse
3. Lesquels des signes de danger la mère peut expliquer à l'ASC?	Diarrhée de plus de 14 jours<br>Diarrhée avec du sang<br>Vomit tout ce qu'il consomme<br>Toux de plus de 14 jours<br>Fièvre de plus de 7 jours
4. Lesquels des signes de danger l'ASC peut observer ?	Incapable de boire ou de téter<br>Sommeil inhabituel ou inconscient<br>Convulsion
5. Lesquels des signes de danger l'ASC peut constater à l'examen physique de l'enfant?	Fièvre<br>Pâleur palmaire<br>Respiration difficile et rapide<br>Raideur de la nuque<br>PB avec coloration rouge avec appétit faible ou Œdèmes bilatraux +++

QUESTIONS RAPIDES

L'enfant présente-t-il un signe de danger ?	Oui	Non	Référer l'enfant
Sam - tousse pendant 2 semaines	X		X
Murat - tousse pendant 2 mois	X		X
La beauté - fait une diarrhée avec présence de sang dans les selles pendant 3 jours	X		X
Marco – a une diarrhée pendant 10 jours		X	
Salimata- a un PB rouge- test appétit bonne		X	
Amina - fièvre pendant 3 jours		X	
Nilgun - fièvre pendant 8 jours		X	
Ida - diarrhée pendant 2 semaines		X	
Tika – a fait une convulsion hier	X		X
Carmen – a une toux pendant 1 mois	X		X
Maria - vomit de la nourriture mais boit de l'eau	X		X
Thomas – n'a rien mangé ni bu à cause des plaies buccales	X		X
Abdoul- PB rouge+ test appétit médiocre	X		X

Insérez votre site web

MODULE 2

TRIAGE ET ADMISSION DE L'ENFANT SOUFFRANT DE MALNUTRITION AIGUE

Module de Formation des Agents de Santé Co mmunautaire sur le Protocole PCIMA

ACTION CONTRE LA FAIM

OBJECTIFS DU MODULE 2

A la fin de ce module, l'agent de santé communautaire (ASC) doit être capable de:

1. Savoir prendre les mesures anthropométriques de l'enfant
2. Maitriser les procédures de triage
3. Admettre un enfant avec une malnutrition aigüe sévère sans complications médicales
4. Référer les enfants avec une malnutrition aigüe sévère avec des complications

CE DONT L'ASC A BESOIN

- **La** disponibilité **d'un dispositif de** lavage des mains **au savon**
- **La** disponibilité de l’eau **potable pour** boire
- **Une balance**
- **Une Bande de Shakir ou MUAC**

LAVAGE DE MAINS AU SAVON

L'ASC doit se laver les mains avec savon

1. Avant de toucher l'enfant,
2. Après avoir touché l'enfant
3. Après avoir salué la mère ou l'accompagnant
4. Avant d'effectuer toute intervention de nettoyage ou d'aseptique,
5. Après exposition aux fluides corporels (urines, sang)

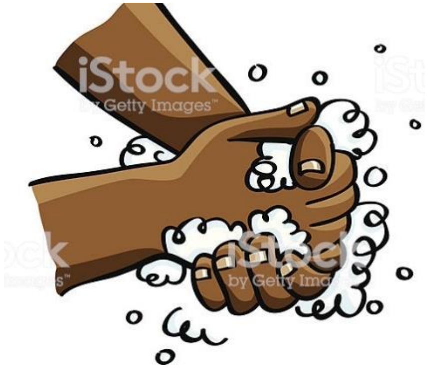

PRENDRE LE PÉRIMÈTRE BRACHIAL (PB)

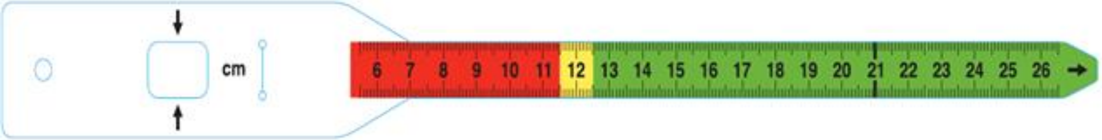

Trouvez, sur le bras de l'enfant, le point central

Apposez l'extrémité la plus large de la bandelette sur ce point central.

Enroulez l'autre extrémité de la bandelette autour du bras de l'enfant

Pas trop séré

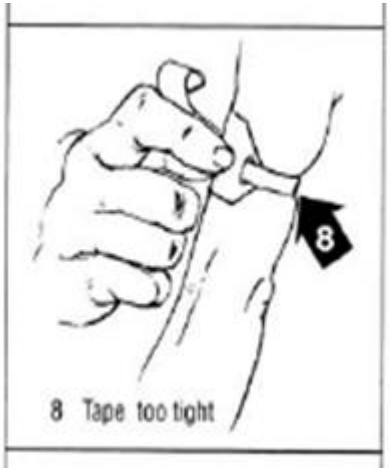

Pas trop lâche

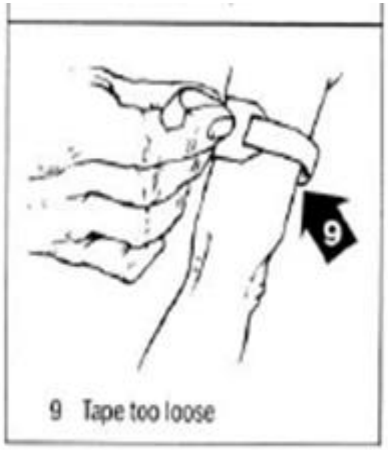

PRENDRE LE PÉRIMÈTRE BRACHIAL (PB)

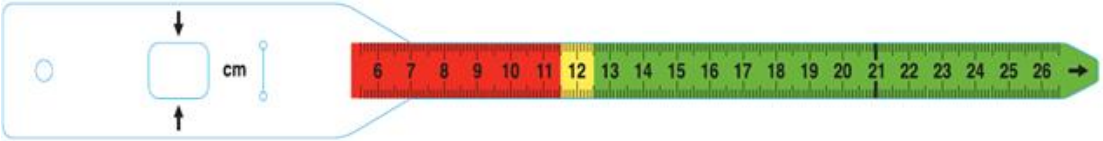

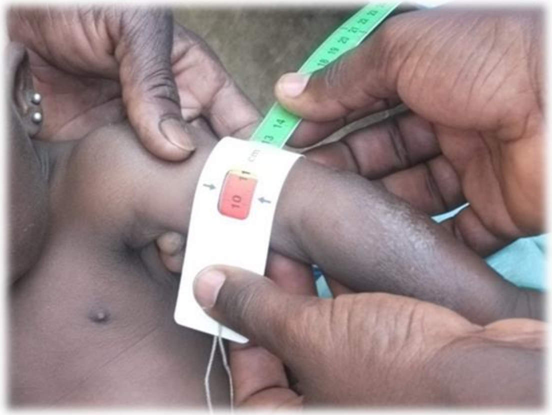

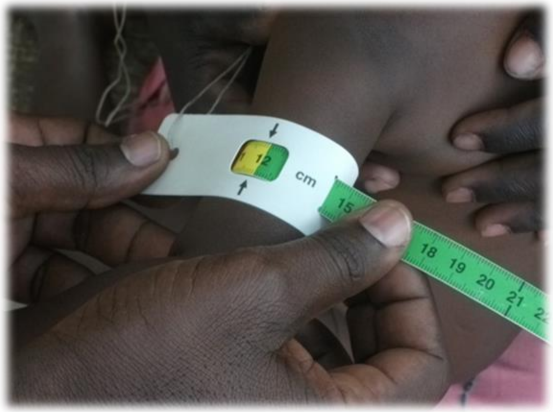

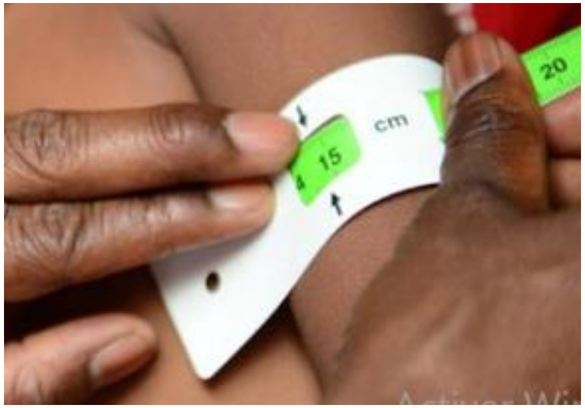

ENFANT MALNUTRITION AIGUE SEVERE < 115mm

ENFANT MALNUTRITION AIGUE MODERE 115-125mm

ENFANT BIEN NUTRI >125mm

VÉRIFIER LES ŒDÈMES BILATÉRAUX

1. On exerce une pression normale avec le pouce sur les deux pieds pendant au moins trois secondes
2. Si l'empreinte du pouce persiste sur les deux pieds

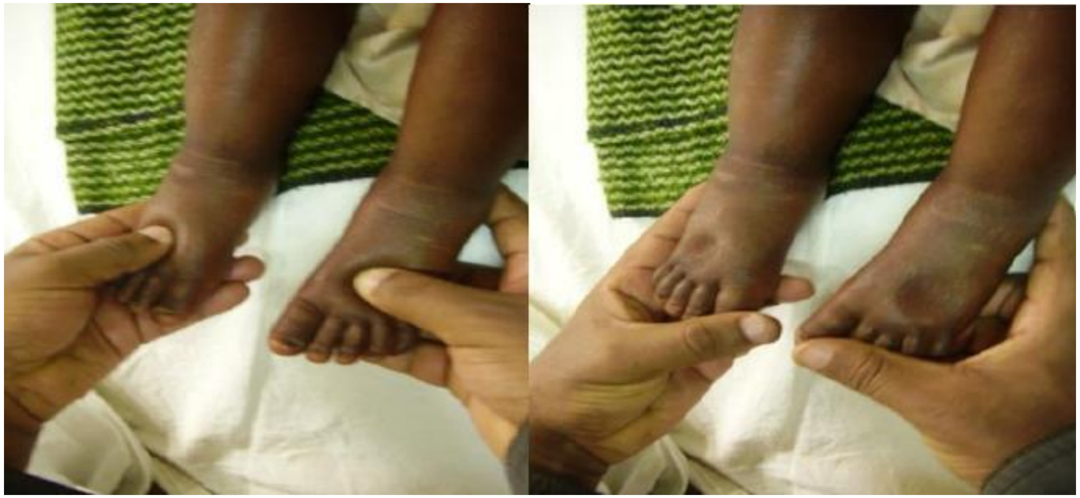

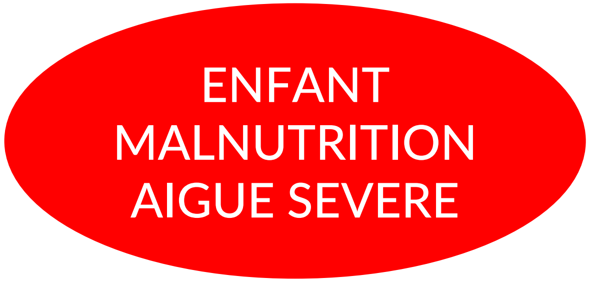

Sévérité des oedèmes Codification

- **Œdèmes Légers +** : des 2 pieds +
- **Œdèmes Modérés ++:** des 2 pieds et la partie inférieure des 2 jambes, ou les 2 mains et la partie inférieure des 2 avant-bras. Intermédiaire entre le degré d'oedèmes légers et sévères
- **Œdèmes Sévères+++** : généralisés soit incluant les 2 pieds, jambes, mains, bras et le visage

PRENDRE LE POIDS

Avec la balance électronique double poids

1. Enfant qui peut marcher, directement sur la balance

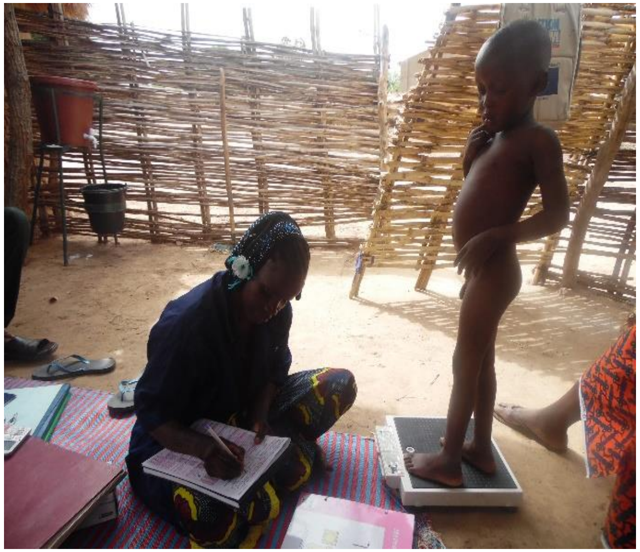

PRENDRE LE POIDS

Avec la balance électronique double poids

2. Enfant qui ne marche pas

- Mettre la balance en marche
- Prendre le poids de la mère
- Mettre la balance à 0, la mère étend sur la balance
- Donner l'enfant à la mère
- Marquer le poids qui se trouve sur l'ecran

Figure 4 Measurement techniques for child weight

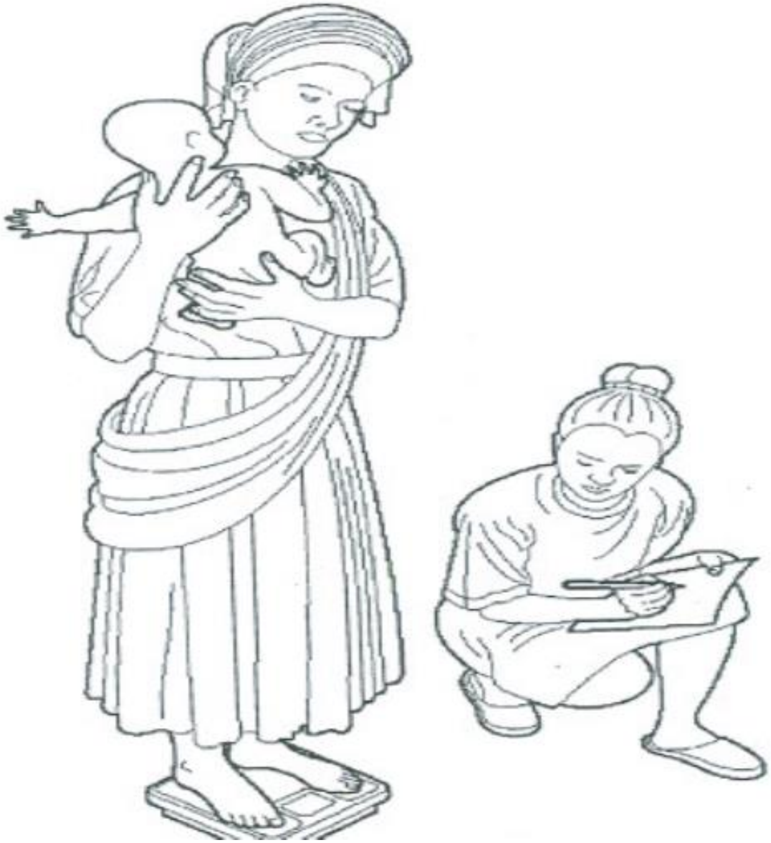

PRENDRE LE POIDS

Avec la balance salter

1. Regarder directament le numero

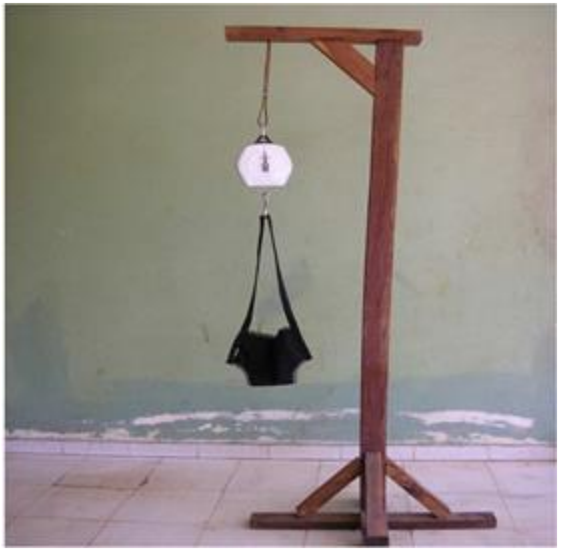

TEST DE L'APPÉTIT

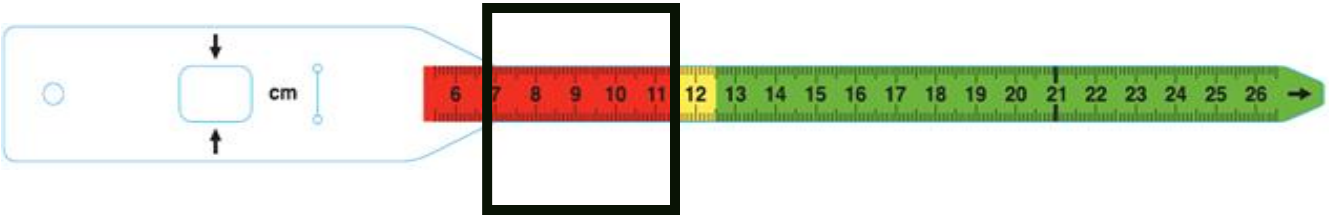

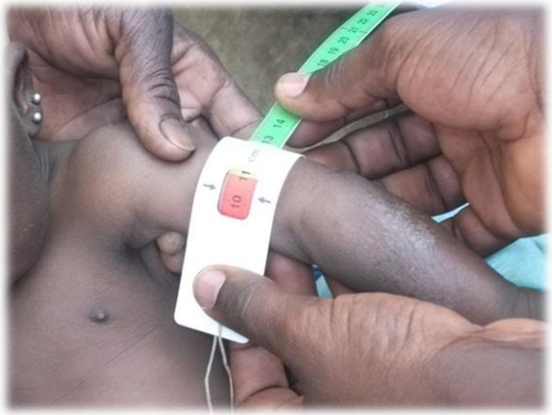

Faire le test de l'appétit à tout enfant avec une malnutrition aigüe sévère ou avec un MUAC rouge

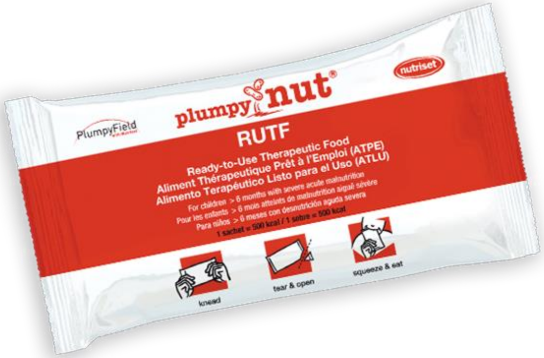

ENFANT MALNUTRITION AIGUE SEVERE

TEST DE L'APPÉTIT

Trouver un endroit calme et un peu à l'écart

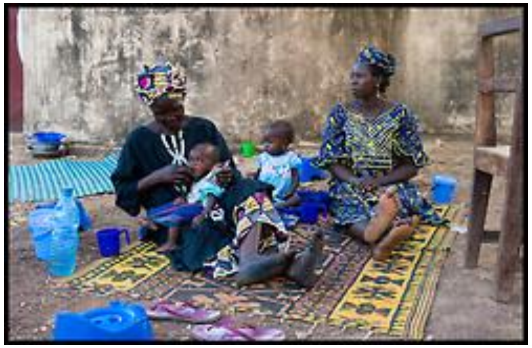

Demander à l'accompganant de laver les mains ainsi que celles de son enfant

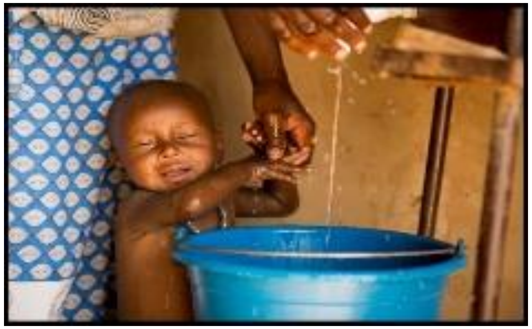

Donner l'ATPE directement avec le sachet, accompagné d'un verre/tasse d'eau potable

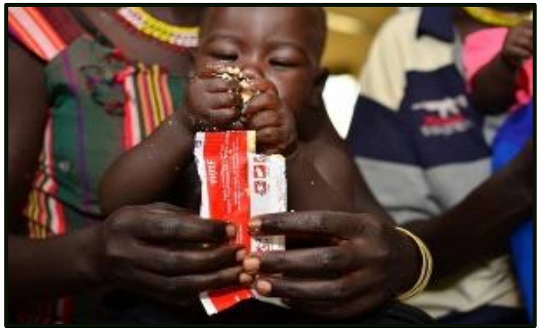

TEST DE L'APPÉTIT

LAVER BIEN LES MAINS DE L'ENFANT ET AUSSI DE LA MÈRE AU ACCOMPAGNANTE

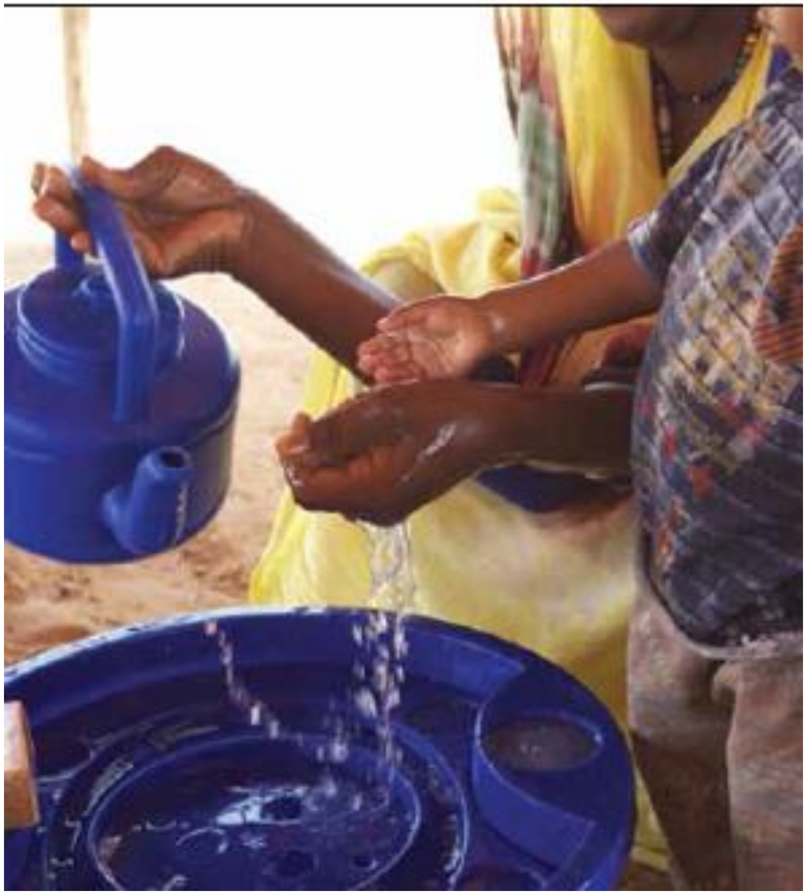

PENDANT LA DURÉE DU TEST DE L'APPÉTIT L'ENFANT DOIT AUSSI BOIRE DE L'EAU POTABLE

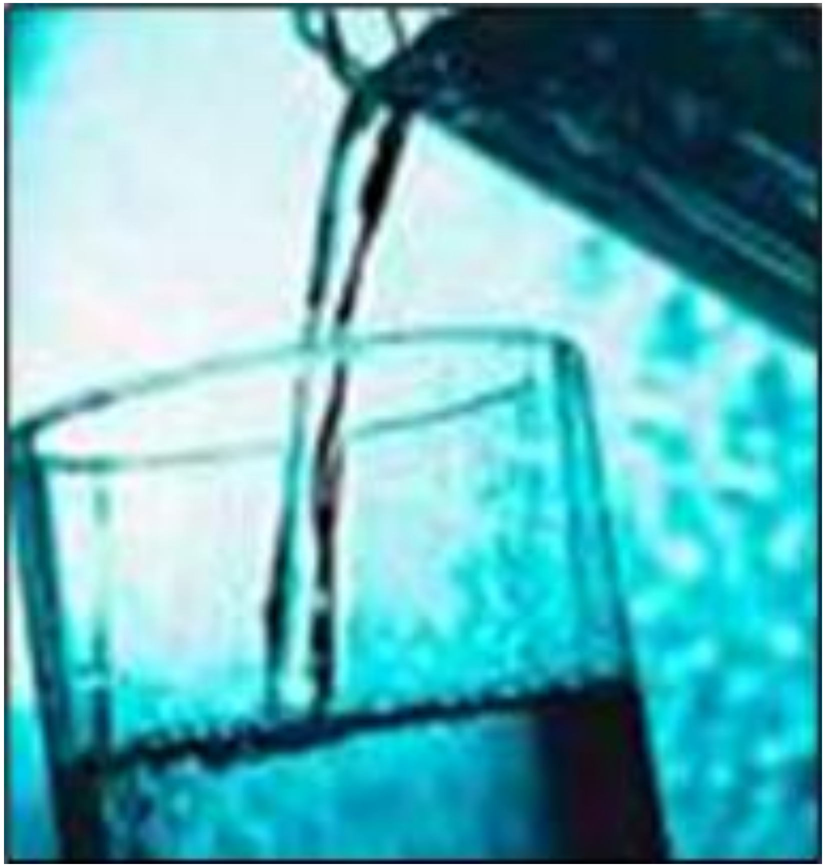

TEST DE L'APPÉTIT

Poids en Kg	Test de l'appétit bon	Matériel
Moins 4 – 6.9	¼ ou plus du contenu	Verre de thé n°8 gradué en 4 parties égales
7 - 9.9	1/3 ou plus du contenu	Verre de thé n°8 gradué en 3 parties égales
10 – 14.9	½ ou plus du contenu	Verre de thé n°8 gradué en 2 parties égales

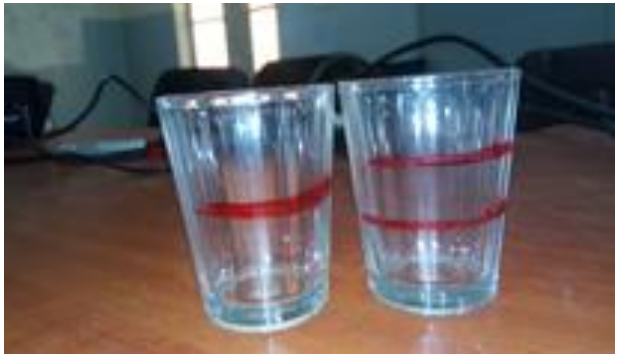

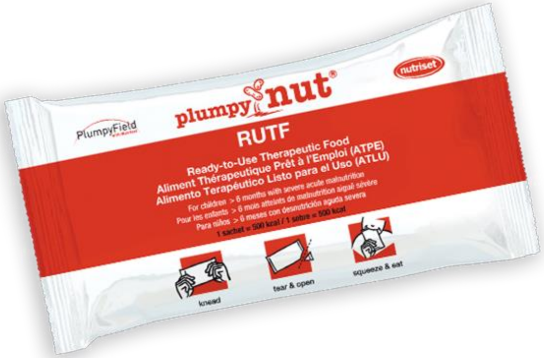

TEST DE L'APPÉTIT

Poids en Kg	Faible	Bon
**Moins 4 – 6.9**	< 1/ 4 sachet	> 1/3 sachet
**7 - 9.9**	< 1/3 sachet	> 1/2 sachet
**10 – 14.9**	< 1/ 2 sachet	> 3/4 sachet

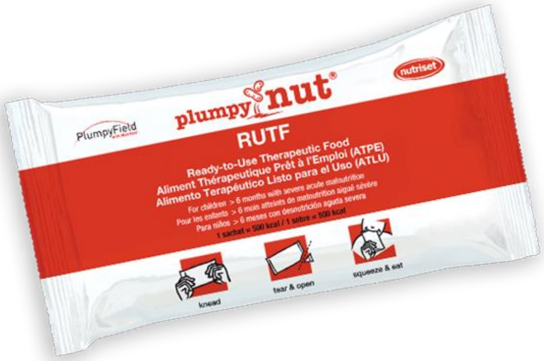

CRITÈRES D'ADMISSION

Type d’admission	Critères
Nouvelle admission	• Age entre 6 -59 mois<br>• PB rouge<br>• P/T< -3<br>• Appétit bon<br>• Œdèmes bilatéraux +, ++<br>• Absence d’autres signes de dangers
Réadmission après abandon < 2 mois	• Retour au site ASC/Case de santé dans les 2 mois après l’abandon du traitement

CAS D’ADMISSION

PB rouge → Sans complications **médicales** → Œdèmes bilatéraux +,++ → Test appétit bon → Admis MAS

PB jeune → Admis MAM

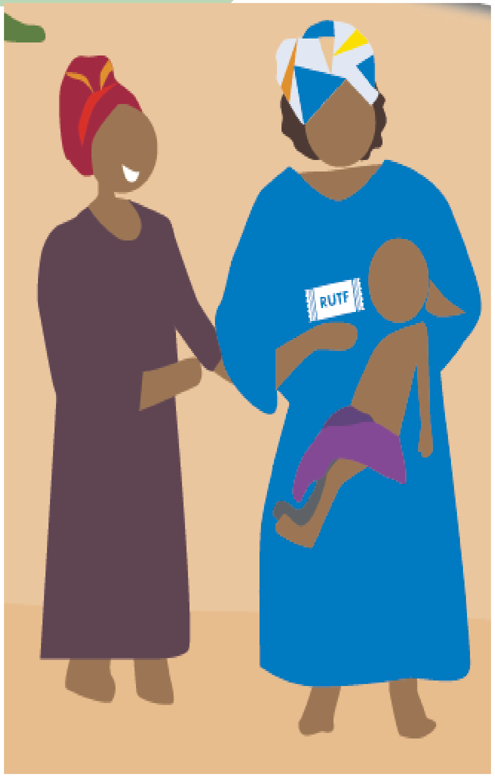

CAS DE RÉFÉRENCEMENT

Enfant → PB rouge → Œdèmes bilatéraux +++ → Test appétit faible → Référer

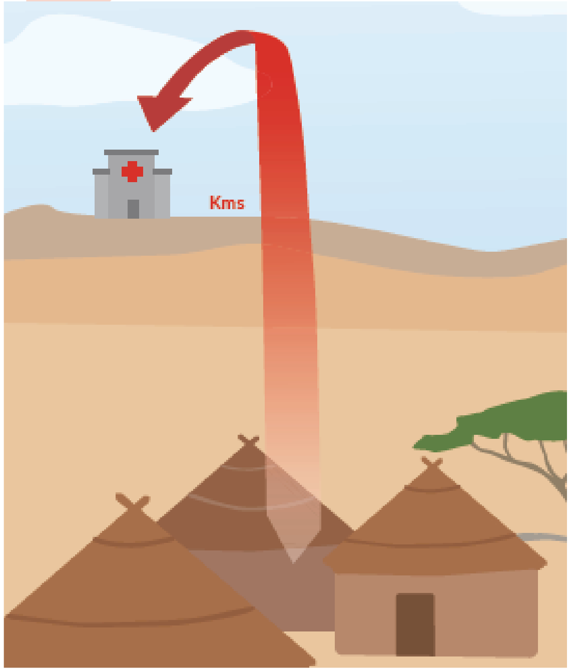

MESSAGES CLÉS

Question	Réponse
1. Quels sont les matériels que l'ASC doit-il avoir pour identifier une malnutrition aigüe chez un enfant ?	• Disponibilité d'un dispositif l'eau pour se lavage des mains au savon<br>• Disponibilité de l'eau potable pour boire<br>• Balance<br>• Périmètre brachial
2. Comment faire le test de l'appétit	• Endroit calme<br>• Laver les mains de l'enfant<br>• Donner ATPE avec beaucoup de l'eau potable
3. Comment identifier un enfant avec une malnutrition aigüe severe sans complications	• PB rouge<br>• Oedèmes bilatéraux +,++<br>• Test de l'appétit

QUESTIONS RAPIDES

L'enfant présente-t-il la malnutrition aigüe sévère ?	Oui	Non	Référer l'enfant	Admis
Sam – PB rouge, sans complications médicales, test de l'appétit faible	X		X	
Hassane- PB vert, 2 mois, vomit tout ce qu'il ingère		X	X	
Murat -sans œdèmes, PB rouge, test de l'appétit bon	X			X
La Beauté –PB jaune, avec toux depuis deux jours		X		X
Marco – œdèmes bilatéraux , PB rouge, diarrhée plus de 2 semaines	X			X
Aminou- œdèmes bilatéraux , PB jaune, toux depuis 1 semaine		X		X
Salimata- PB rouge, test appétit bon, fièvre depuis 2 jours	X			X

Insérez votre site web

MODULE 3

TRAITEMENT DE L'ENFANT SOUFFRANT DE MALNUTRITION AIGÜE SÉVÈRE SANS COMPLICATIONS MÉDICALES

Module de Formation des Agents de Santé Communautaire sur le Protocole PCIMA

ACTION CONTRE LA FAIM

OBJECTIFS DU MODULE 3

A la fin de ce module, l'agent de santé communautaire (ASC) doit être capable de:

1. Administrer le traitement systématique aux enfants malnutris aigüe sévère sans complications médicales
2. Administrer le traitement nutritionnel MAM et MAS

CE DONT L'ASC A BESOIN

La disponibilité **d'un dispositif de** lavage des mains **au savon**

Un thermomètre

Une balance

Une Bande de Shakir ou MUAC

TRAITEMENT

TRAITEMENT NUTRITIONNEL	MALNUTRITION AIGUE	
	MAM<br>Sachet par semaine<br>ASPE	MAS<br>Sachet par semaine<br>ATPE
**3,0 – 3,4 Kg**	7 sachet/semaine	8 sachet /semaine
**3,5 – 4,9 Kg**		10 sachet /semaine
**5.0 – 6.9 Kg**		15 sachet /semaine
**7.0 – 9.9 Kg**		20 sachet /semaine
TRAITEMENT SYSTEMATIQUE		Amoxicilline, mebendazol, vaccine rougeole

A L'ADMISSION. SEMAINE 1.MAS

	Amoxicilline comprimé 250 mg	
Poids	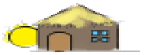 Matin	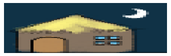 Soir
<5kg 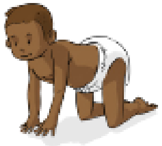	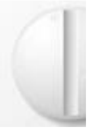	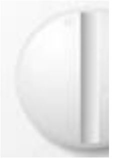
5-10 Kg 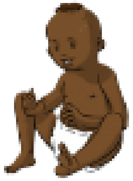	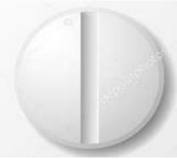	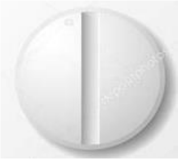
10-20 kg 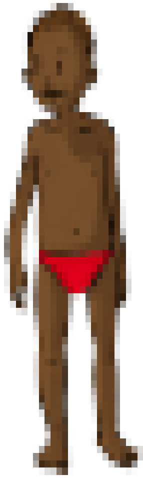	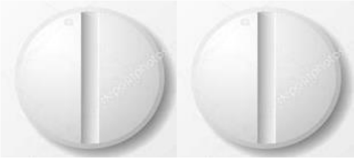	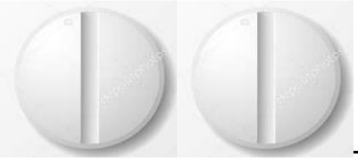
20-35 kg	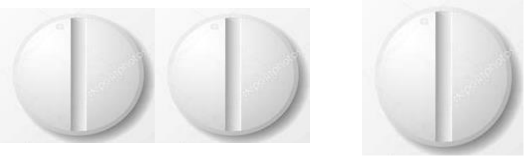	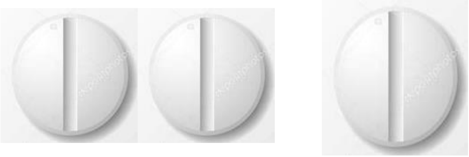
> 35 Kg	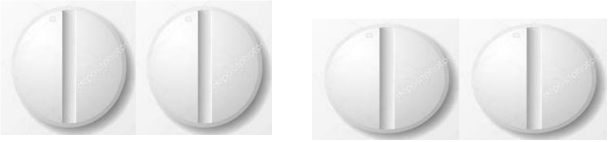	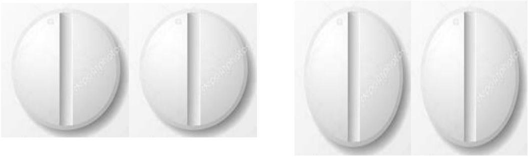

SEMAINE 2. MAS

Déparasitage, seulement pour les enfants qui peuvent marcher

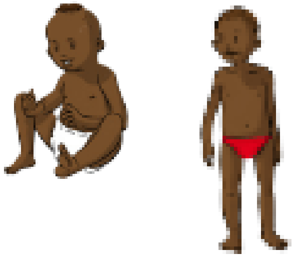	< 1 an	1-2 ans	> = 2 ans
**Albendazole 400 mg**	Ne pas administrer	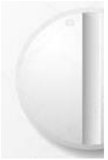	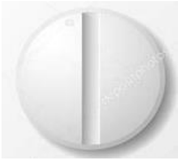
**Mébendazole 500 mg**	Ne pas administrer	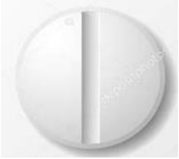	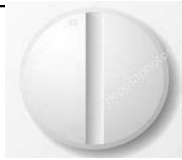

SEMAINE 4. MAS

Vérifier si l'enfant a reçu le vaccin anti- rougeoleux

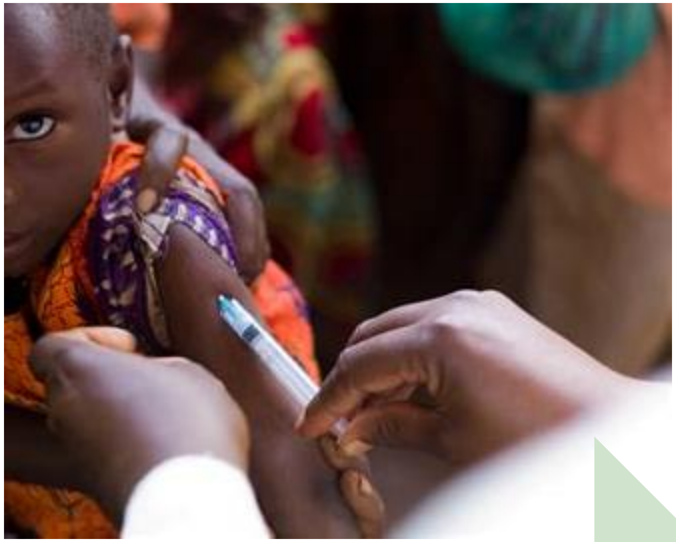

Enfant à la 4ème visite → **Pas vacciné contre la** rougeole → Contacter/**référer** l'équipe **mobile** du centre de santé

CHAQUE SEMAINE.MAS

Enfant avec **une fièvre**

Prendre **la température**

Test diagnostique rapide +

Donner **du Coartem**

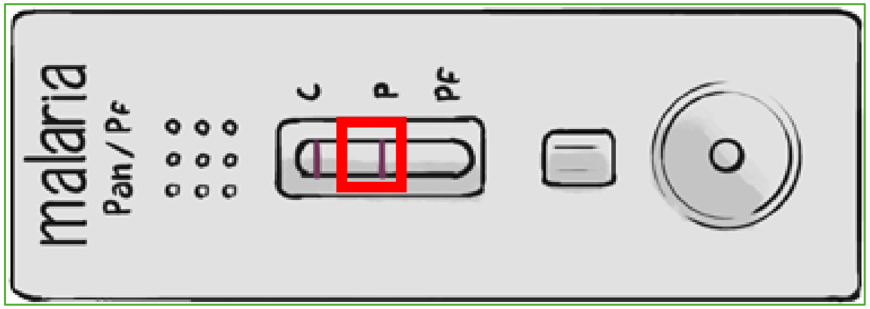

CHAQUE VISITE SI TDR +

COARTEM

	COARTEM 1 (COMPRIME = 20 mg AM et 120 mg LM)					
Poids	Jour1		Jour2		Jour3	
< 14kg	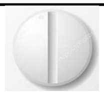	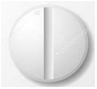	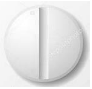	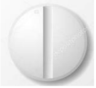	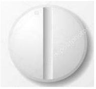	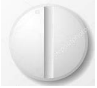
14-25 kg	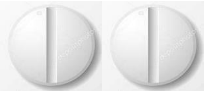	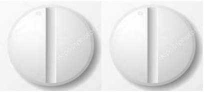	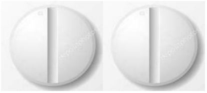	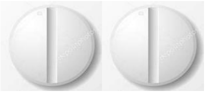	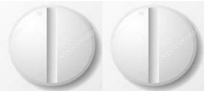	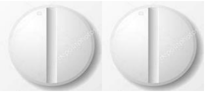

CHAQUE VISITE SI TDR +

COARTEM

	COARTEM (Suspension 5 ml=15 mg AM + 90 mg LM)		
Poids	Jour1	Jour2	Jour3
5kg	7 ml	7 ml	7 ml
7.5 kg	10 ml	10 ml	10 ml
10 kg	14 ml	14 ml	14 ml
15 kg	20 ml	20 ml	20 ml

CHAQUE SEMAINE. MAS

	ATPE	
Poids	sachet par jour	sachet par semaine
3,0 – 3,4 Kg 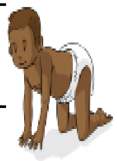	1 ¼ 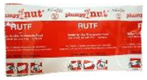	8 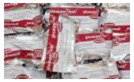
3,5 – 4,9 Kg	1 ½ 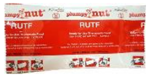	10 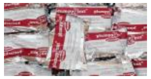
5.0 – 6.9 Kg 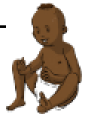	2 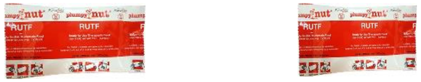	15 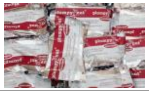
7.0 – 9.9 Kg 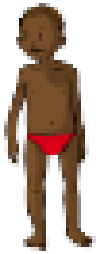	3 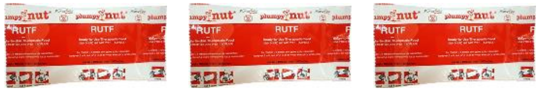	20 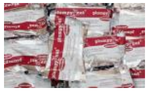
10.0 – 14.9 Kg	4 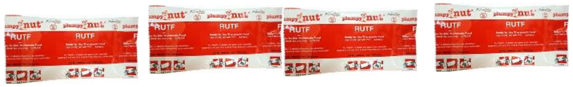	30 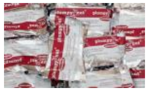
15.0 – 19.9 Kg	5 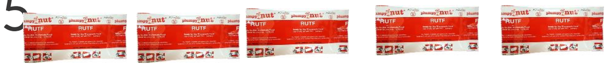	35 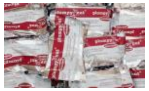
20.0 – 29.9 Kg	6 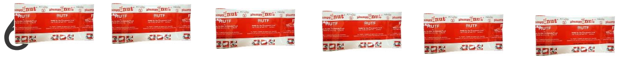	40 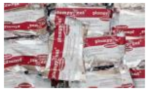
30.0 – 39.9 Kg	7 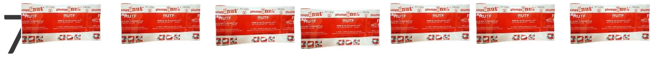	50 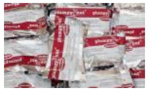
40 – 60 Kg	8 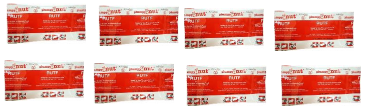	55 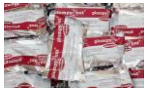

CHAQUE SEMAINE. MAS

	ATPE	
Poids	sachet par jour	sachet par semaine
3,0 – 3,4 Kg 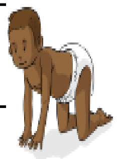	1 ¼ 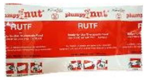	8 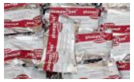
3,5 – 4,9 Kg	1 ½ 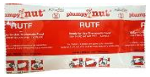	10 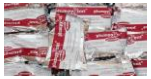
5.0 – 6.9 Kg 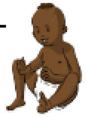	2 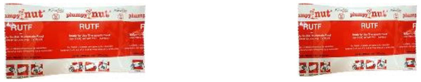	15 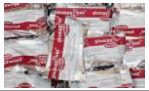
7.0 – 9.9 Kg 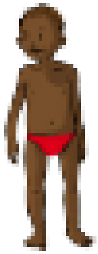	3 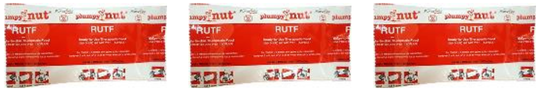	20 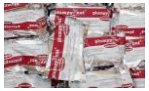
10.0 – 14.9 Kg	4 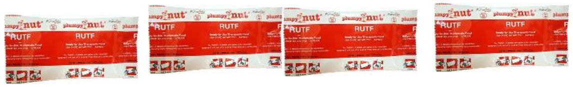	30 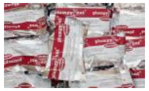
15.0 – 19.9 Kg	5 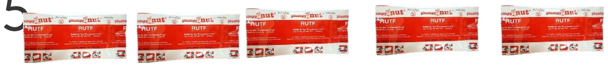	35 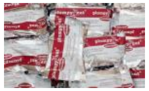
20.0 – 29.9 Kg	6 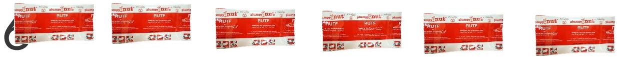	40 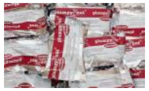
30.0 – 39.9 Kg	7 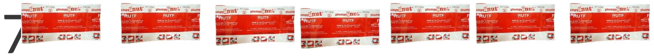	50 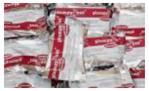
40 – 60 Kg	8 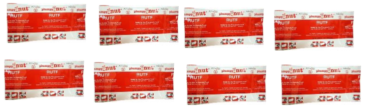	55 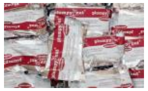

MESSAGES CLÉS

Question	Réponse
1. Quelles sont les médicaments que l'ASC doit-il avoir pour les enfants MAS sans complications médicales ?	• Amoxicilline<br>• Paracétamol<br>• Mebendazole/albendazole<br>• Coartem
2. Comment calcule –t-on les quantités d'ATPE à donner à l'enfant?	• Avec le poids
3. Conseils à la mère pour prendre l'ATPE à la maison	• Toujours allaiter avant de donner l'ATPE<br>• Lavage les mains de l'enfant au savon<br>• Prendre beaucoup d'eau potable<br>• Ne pas partager l'ATPE avec les autres enfants

QUESTIONS RAPIDES

	Vrai	Faux
Sam – PB rouge, sans complications médicales. L' ASC lui a donné Coartem à l'admission		X
Hassane- PB rouge sans complications, sans fièvre, l'ASC lui a donné amoxicilline et ATPE le jour de l'admission	X	
Murat –PB rouge sans complications, avec fièvre et TDR+, l'ASC a donné du coartem et ATPE à la 3ème semaine du suivi	X	
La Beauté – PB rouge, test de l'appétit faible. L'ASC a donné 10 sachets de ATPE à la troisième visite		X
Marco – A la semaine 4, l'enfant avec malnutrition aigüe, doit recevoir le vaccin contre la tuberculose		X
Rosine- PB rouge, à partir de la 5 ème semaine a partagé l'ATPE avec sa petite soeur		X
Aminou- durant toute la durée du traitement de la MAS a reçu amoxicilline, mebendazole et ATPE	X	
Salimata-PB rouge, 2ème visite pour le suivi, l'ASC lui a donné du mebenadazole et ATPE	X	

Insérez votre site web

MODULE 4

SUIVI HEBDOMADAIRE DE L'ENFANT SOUFFRANT DE MALNUTRITION AIGUE

Module de Formation des Agents de Santé Communautaire sur le Protocole PCIMA

ACTION CONTRE LA FAIM

OBJECTIFS DU MODULE 4

A la fin de ce module, l’agent de santé communautaire (ASC) doit être capable de:

1. Connaitre les actions et traitement qu’il faut faire a chaque visite
2. Identifier quand il faut référer l’enfant
3. Conseilles à la mère

CE DONT L'ASC A BESOIN

- **la** disponibilité **d'un dispositif** lavage des mains **au savon**
- **Un** thermomètre
- **Une balance**
- **Une Bande de Shakir ou MUAC**

LAVAGE DES MAINS AU SAVON

L'ASC doit se laver les mains avec du savon

1. Avant de toucher l'enfant,
2. Après avoir touché l'enfant
3. Après avoir salué la mère ou l'accompagnant
4. Avant d'effectuer toute intervention de nettoyage ou d'aseptique,
5. Après exposition aux fluides corporels (urines, sang)

SUIVI DE L'ENFANT À CHAQUE VISITE

1

Accueillir la mère

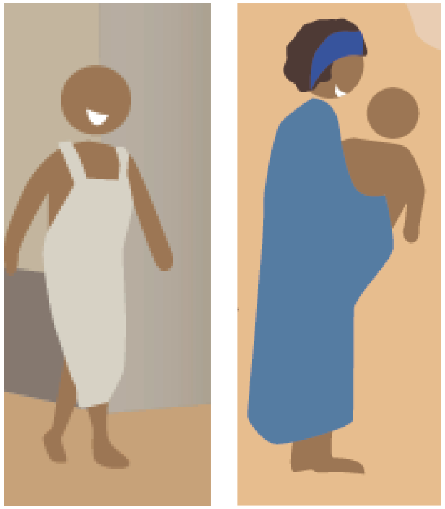

Se laver les mains

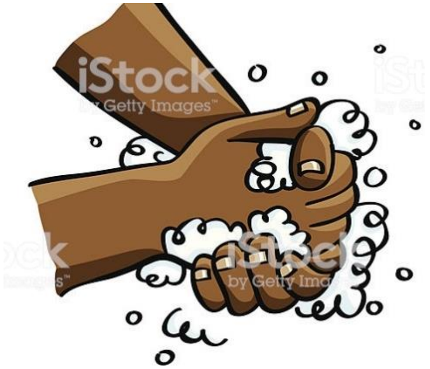

SUIVI DE L'ENFANT À CHAQUE VISITE

2

Identifier **les signes de** danger (Module 1)

- Ecouter la mère
- Observer l'enfant
- **Examen physique de l'enfant**

SUIVI DE L'ENFANT À CHAQUE VISITE

3

Mesures anthropométriques (Module 2)

Mesure PB

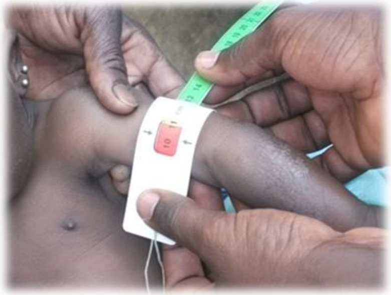

Prendre le Poids

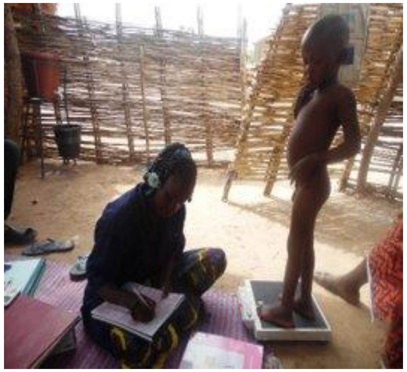

Recherche **des œdèmes bilatéraux**

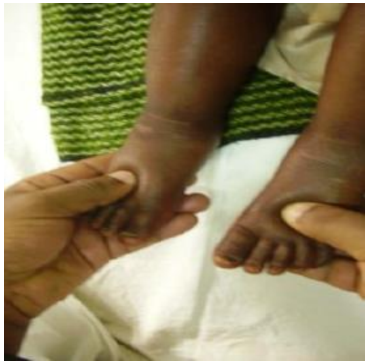

SUIVI DE L’ENFANT À CHAQUE VISITE

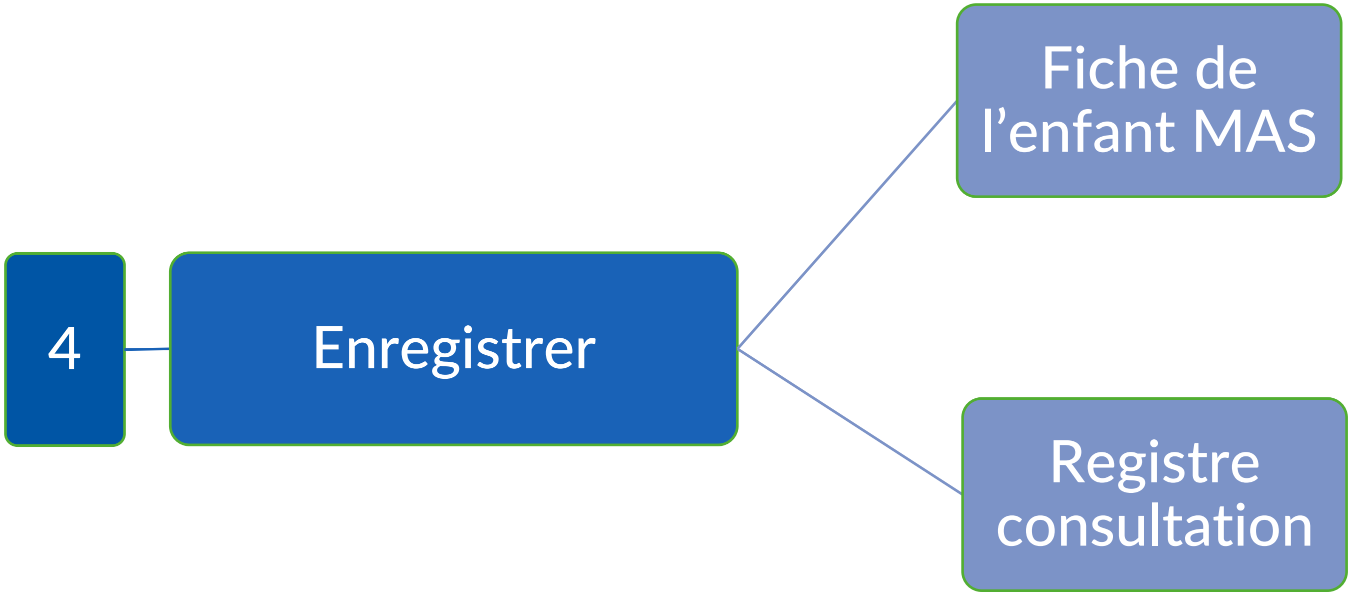

ARBRE DE DÉCISION

Signes de danger	**Référer** l'enfant	Module 1
MUAC **jaune** ou rouge	**On peut continuer avec le** Traitement nutritionnel	Module **3**
MUAC vert	**Décharger** l'enfant	Module 5

SUIVI ENFANT MAM

1. Suivi tous les 15 jours
2. Suivi avec le poids et PB a chaque visite
3. Mebendazol la première visite
4. 14 sachet de ASPE a chaque visite

SUIVI HEBDOMADAIRE MAS

	Sem 1 Adm	Sem 2	Sem 3	Sem 4	Sem 5	Sem 6	Sem 7	Sem 8
Signes de danger	✓	✓	✓	✓	✓	✓	✓	✓
PB	✓	✓	✓	✓	✓	✓	✓	✓
Poids	✓	✓	✓	✓	✓	✓	✓	✓
Test appetit	✓	A toute visite s'il y a poids et/ou PB stationnaire						
Traitement nutritionnel	✓	✓	✓	✓	✓	✓	✓	✓
Traitement systematique	Amoxi ✓	Mebendazol ✓		Vaccine rougeole ✓				
TDR, coartem	A toute visite s'il y a la fièvre, on fait le TDR, si TDR+, coartem							

CONSEILS CHAQUE VISITE MAS

1. Sensibiliser la mère que l'enfant doit toujours être **allaité** et à la demande avant qu'on lui donne des ATPE
2. L'**ATPE** est une nourriture et un **médicament** destiné exclusivement aux enfant malnutris, à ne pas partager aux autres enfants à la famille
3. L'enfant ne doit jamais être forcé à manger

CONSEILS CHAQUE VISITE. MAS

5. Laver les mains de l’enfant ainsi que son visage avec du savon avant de le nourrir et donner l’ATPE

6. L'enfant doit prendre l’ATPE avec beaucoup d’eau potable

QUESTIONS RAPIDES

Question	Réponse
1. Quelles sont les actions qu'il faut faire a chaque visite?	• Identifier signes de danger<br>• Poids, PB et œdèmes<br>• Test de l'appétit s'il n'y a pas l'augmentation de poids<br>• TDR s'il y a fièvre
2. A quels moments l'ASC doit faire le test de l'appétit	A la première visite, et toujours s'il n'y a pas l'augmentation du poids et/ou PB
3. Quelles sont les conseilles a donner à mère?	• Toujours allaiter avant de donner l'ATPE<br>• Lavage les mains de l'enfant au savon<br>• Prendre beaucoup d'eau potable<br>• Ne pas partager l'ATPE avec les autres enfants

CAS PRATIQUE

	Oui	Non
Sam – arrive pour la deuxième visite de suivi. On lui donne l'amoxiciline.		X
Hassane- PB rouge		X
Murat -sans œdèmes, PB rouge, test de l'appétit bon	X	
La beauté –PB jaune, avec toux depuis deux jours		X
Marco – œdèmes bilatéraux , PB rouge, diarrhée plus de 2 semaines	X	
Aminou- œdèmes bilatéraux , PB jaune, toux depuis 1 semaine		X
Salimata- PB rouge, test appétit bon, fièvre depuis 2 jours	X	

Insérez votre site web

MODULE 5

PROCEDURES DE DÉCHARGE DE L'ENFANT ET CONSEILS NUTRITIONNELS

Module de Formation des Agents de Santé Communautaire sur le Protocole PCIMA

ACTION CONTRE LA FAIM

OBJECTIFS DU MODULE 5

A la fin de ce module, l'agent de santé communautaire (ASC) doit être capable de:

1. Maitriser les critères de sortie de l'enfant au programme
2. Donner des Conseils nutritionnels aux mères et/ou accompagnantes

CE DONT L'ASC A BESOIN

- **La** disponibilité **d'un dispositif de** lavage des mains **au savon**
- **Un** thermomètre
- **Une balance**
- **Une Bande de Shakir ou MUAC**

LAVAGE DES MAINS AU SAVON

L'ASC doit se laver les mains avec du savon

1. Avant de toucher l'enfant,
2. Après avoir touché l'enfant
3. Après avoir salué la mère ou l'accompagnant
4. Avant d'effectuer toute intervention de nettoyage ou aseptique,
5. Après exposition aux fluides corporels (urines, sang)

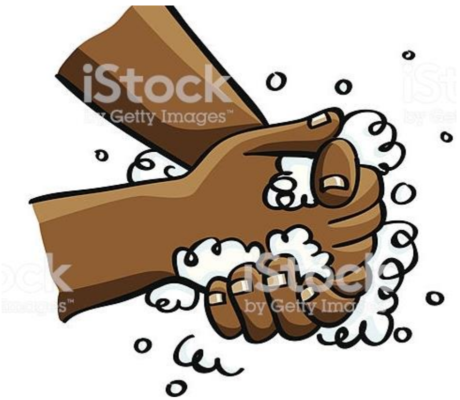

CRITÈRES DE SORTIE MAM

**Guérison**	PB supérieur ou égal à 125 mm (PB vert) pendant 2 visites consécutives<br>P/T > 1,5
**Abandon**	L'enfant n'est pas venu pendant 2 visites consécutives (1 mois)
**Non réponse**	• Non atteinte des critères de sortie après 3 mois<br>• Absence de gain de poids après 6 semaines ;<br>• Perte de poids pendant plus de 4 semaines dans le programme

CRITÈRES DE SORTIE MAS

**Guérison**	PB supérieur ou égal à 125 mm (PB vert) pendant 2 visites consécutives<br>P/T > 1,5<br>Et<br>Absence d'œdèmes nutritionnels pendant 14 jours

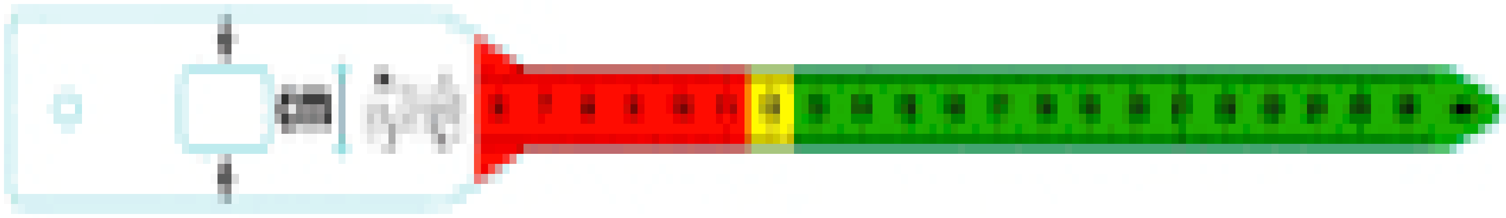

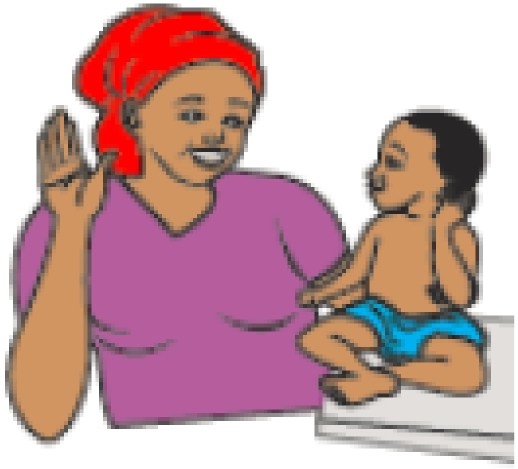

TYPE DE SORTIE MAS

**Guéri**	Enfant avec PB vert
**Abandon**	L'enfant n'est pas venu pendant 2 visites consécutives
**Référence**	Référence vers le CRENI pour non réponse au traitement ou signes de danger
**Décès**	Enfant qui est décédé durant le traitement avec l'ASC
**Transfert**	Transfert vers un autre site de distribution CRENAS (il ne reviendra pas mais est toujours enregistré dans le programme)

NON RÉPONSE AU TRAITEMENT MAS

Perte de poids depuis l'admission	7 jours
Perte de poids (enfants non-œdémateux) durant 2 visites consécutives	Toute visite
Echec à prendre du poids (non-œdémateux)	14 jours
Pas d'amorce de la fonte des œdèmes	14 jours
Echec au test de l'appétit	Toute visite
Œdèmes encore présents	21 jours

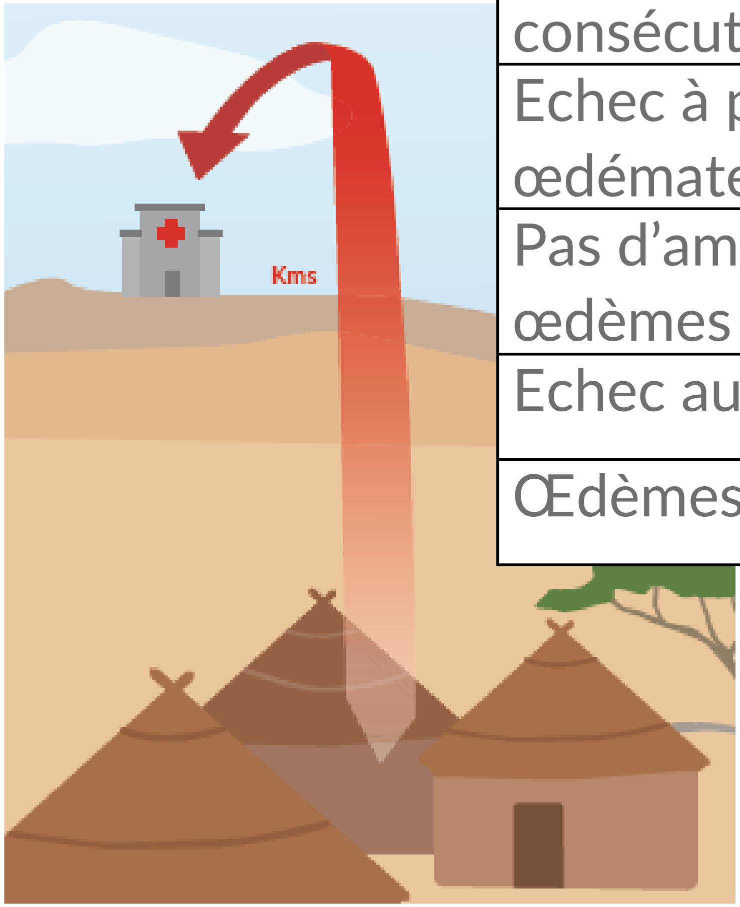

CONSEILS NUTRITIONNELS À DONNER À LA MÈRE À LA DÉCHARGE

1. Encourager les mères à continuer l’allaitement;
2. Appuyer, encourager et renforcer les pratiques recommandées d’alimentation ;
3. Appuyer, encourager et renforcer les pratiques d'alimentation de complément recommandées en utilisant les aliments disponibles localement ;
4. Encourager les visites mensuelles de suivi de croissance ;
5. Améliorer les comportements liés à la santé ;
6. Encourager les mères à participer aux groupes de soutien à l'ANJE ;

MESSAGES CLÉS

Question	Réponse
1. quels sont les types de sortie	• Guéri<br>• Abandon<br>• Non- réponse: référé<br>• Décès<br>• Transfert
2. Cas sortie guéri	• Enfant avec PB vert pendant deux visites consécutives
3. Quelles sont les cas de non réponse au traitement	• Perte de poids depuis l'admission<br>• Perte de poids durant 2 visites consécutives<br>• Echec à prendre du poids 14 jours<br>• Echec au test de l'appétit<br>• Pas d'amorce de la fonte des œdèmes<br>• Œdèmes encore présents

QUESTIONS RAPIDES

**L'enfant présente-t-il la malnutrition aigüe sévère ?**	**Oui**	**Non**	**Référer l'enfant**	**Admis**
Sam – PB rouge, sans complications médicales, test de l'appétit faible	X		X	
Hassane- PB vert, 2 mois, vomit tout ce qu'il ingère		X	X	
Murat -sans œdèmes, PB rouge, test de l'appétit bon	X			X
La beauté –PB jaune, avec toux depuis deux jours		X		X
Marco – œdèmes bilatéraux , PB rouge, diarrhée plus de 2 semaines	X			X
Aminou- œdèmes bilatéraux , PB jaune, toux depuis 1 semaine		X		X
Salimata- PB rouge, test appétit bon, fièvre depuis 2 jours	X			X

Insérez votre site web

MODULE 6

REPORTAGE

Module de Formation des Agents de Santé Communautaire sur le Protocole PCIMA

ACTION CONTRE LA FAIM

OBJECTIFS DU MODULE 6

A la fin de ce module, l’agent de santé communautaire (ASC) doit être capable de comprendre et remplir:

1. Remplir le registre CRENAS
2. Remplir les fiches individuelles de suivi de l'enfant MAS (CRENAS)
3. Remplir la Fiche de transfert et le rapport mensuel
4. Notification des cas

MÉTHODE

Ce module, sera entièrement en pratique.

1. Chaque participant va recevoir des copies en papiers du registre CRENAS, fiche individuelle de suivi l'enfant MAS, fiche de transfert
2. Le travail sera fait en groups de deux personnes
3. Le formateur va partager les cas cliniques
4. Le formateur va faciliter le travail par groupe

CAS CLINIQUE

Cas clinique

Mamadou Traoré, 8 mois arrive au site ASC le 23 mars 2020. Prénom et Nom de la mère: Salimata Sangaré , Prénom du père :Aminou.

Adresse : Korofina Sud

A l'admission

Pas de pâleur palmaire , respiration normale, pas de fièvre,
poids : 4, 200 kg; PB: 113mm, pas d'œdèmes bilatéraux

4 -ème Visite

Diarrhée avec du sang durant 1 semaine
Poids: 4, 300 kg, PB: 114mm, pas d'œdèmes bilatéraux

Sortie

Transfer au centre de santé

REGISTRE ENFANT MAM

No d'ordre	Prénom et Nom	Adresse et N° Tél.	NC/AC	Enfants			Femmes		Admission						Vitamine A		Alben/Mében		Fer-acide fol	
				Suivi ex MAS	Age (mois)	Sexe(M/F)	FE/FA	Age(ans)	Date	Poids (kg.g)	Taille (cm)	PB (mm)	P/T ou IMC	Poids cible	Date	Dose	Date	Dose	Date	Dose

Admission
Ne pas oublier le PB poids et les oedèmes

REGISTRE ENFANT MAM

Ration		viste 1					visite 2					Visite 3					visite 4				
Qtité	Type	Date	PB (mm)	Poids (kg.g)	Ration		Date	PB (mm)	Poids (kg.g)	Ration		Date	PB (mm)	Poids (kg.g)	Ration		Date	PB (mm)	Poids (kg.g)	Ration	
					Qtité	Type				Qtité	Type				Qtité	Type				Qtité	Type

Visite 5						Visite 6					Visite 7					Visite 8					Sorties									Stimulation psycho cognitive
Date	PB (mm)	Poids (kg.g)	Taille (cm)	Ration		Date	PB (mm)	Poids (kg.g)	Ration		Date	PB (mm)	Poids (kg.g)	Ration		Date	PB (mm)	Poids (kg.g)	Ration		Date	PB (mm)	Poids (kg.g)	Taille (cm)	P/T ou IMC	Poids cible	Ration		Type de sortie(guéri, abandon, déces, référence/non répondant, suivi ex mas)	
				Qtité	Type				Qtité	Type				Qtité	Type				Qtité	Type							Qtité	Type		

A toute visite, PB , poids et ASPE

REGISTRE ENFANT MAS

Admission dans la structure													
	TYPE D'ADMISSION												
Mode d'Admission	NC	Rechute	Réadmission	TN / TM	Sex F/M	Age mois	PB mm	Poids kg,g	Taille cm	P/T ou IMC	Œd. 0,+,++,+++	pathologie associées

Admission
Spontanée
Dépistage actif

Admission
Ne pas oublier le PB poids et les oedèmes

REGISTRE ENFANT MAS

ANNEXES

Sortie						Type de Sortie					Date du Poids Minimum	Poids Minimum	Seances stimulation psychocognitive	Seances CCSC	Observations
Date jj/mm	Poids kg,g	Taille cm	P/T ou IMC	Œd. 0,+,++,+++	PB mm	guéri	décès	abandon	Non repondant	trans/reréfér Nut/Med					

Décharge
Type de sortie

Décharge
Mesures anthropométriques à la décharge

FICHE INDIVIDUELLE DE SUIVI DE L'ENFANT MAS

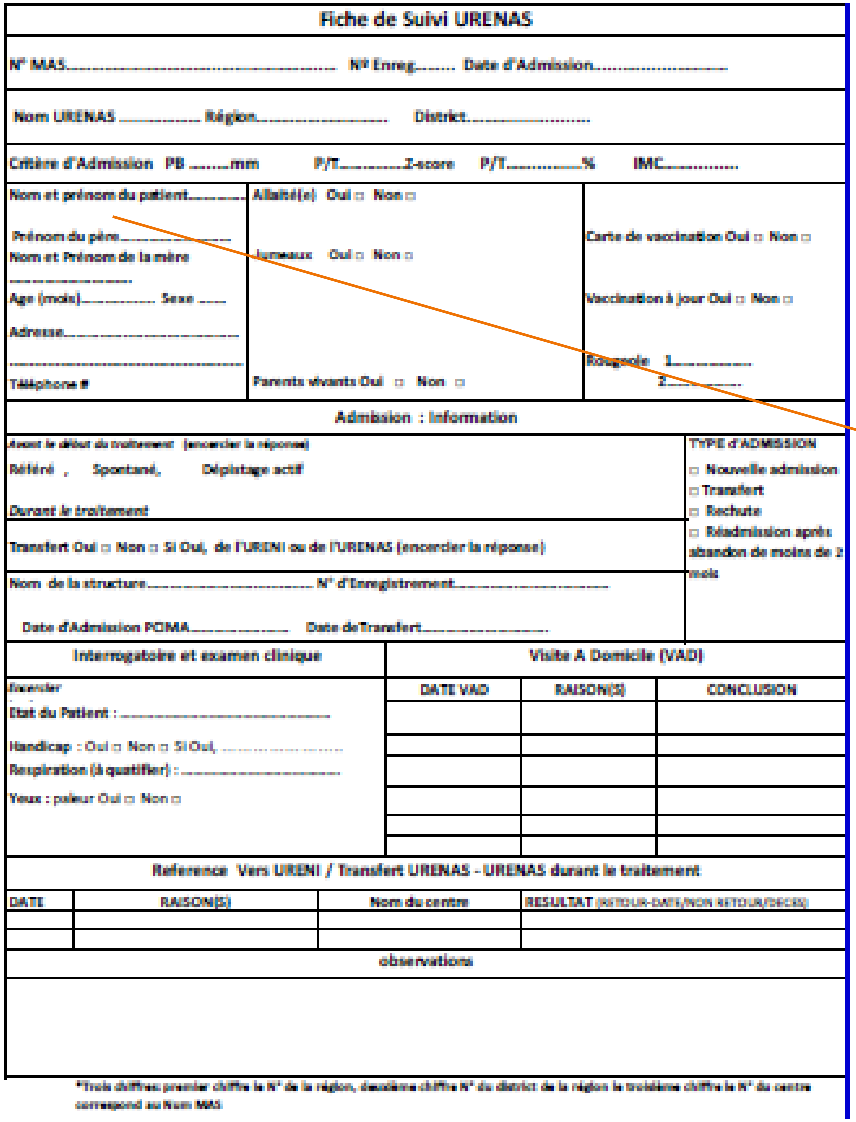

Fiche de Suivi URENAS

N° MAS.. Nº Enreg........ Date d'Admission..........................

Nom URENAS .... Région......................... District.........................

Critère d'Admission PB ....mm P/T............Z-score P/T..............% IMC.............

Nom et prénom du patient.......... Prénom du père.................. Nom et Prénom de la mère .... Age (mois)............. Sexe ..... Adresse.................................. .... Téléphone #	Allaité(e) Oui □ Non □ Jumeaux Oui □ Non □ Parents vivants Oui □ Non □	Carte de vaccination Oui □ Non □ Vaccination à jour Oui □ Non □ Rougeole 1............... 2..............

Admission : Information

Avant le début du traitement (encercler la réponse) Référé , Spontané, Dépistage actif *Durant le traitement*	TYPE d'ADMISSION □ Nouvelle admission □ Transfert □ Rechute □ Réadmission après abandon de moins de 2 mois
Transfert Oui □ Non □ Si Oui, de l'URENI ou de l'URENAS (encercler la réponse)	
Nom de la structure.............................. N° d'Enregistrement.............................. Date d'Admission PCIMA................. Date deTransfert......................	

Interrogatoire et examen clinique	Visite A Domicile (VAD)		
Encercler	DATE VAD	RAISON(S)	CONCLUSION
Etat du Patient :			
Handicap : Oui □ Non □ Si Oui, ...			
Respiration (à quatifier) :			
Yeux : paleur Oui □ Non □			

Reference Vers URENI / Transfert URENAS - URENAS durant le traitement

DATE	RAISON(S)	Nom du centre	RESULTAT (RETOUR-DATE/NON RETOUR/DECES)

observations

*Trois chiffres premier chiffre le N° de la région, deuxième chiffre N° du district de la région le troisième chiffre le N° du centre correspond au Num MAS

Données sur l'affiliation de l'enfant

FICHE DE L'ENFANT MAS

Admission : Taille Couchée/Debout..............cm Poids Cible.............kg

Semaines	Adm	2	3	4	5	6	7	8				
Date (j/mm)												
Poids (kg,g)												
PB (mm)												
Test App. (Bon/Moyen/Faibl												
Diarrhées (0 à Nb jrs)												
Vomit (0 à Nb jrs)												
Fièvre (0 à nb jrs)												
Toux (0 à nb jrs)												
Dermatoses												
Conj. Pâles (0 à ++)												
Respirations /min												
Temp. C° (Axi/Rect.)												
Résultat Test Palu (0/-/+)												
ATPE (Nb sachets donnés)												
Séances d'éducation nutritionnelles reçues												
Séances de stimulation psychocognitive												

Médicaments de Routine

Médicaments	Date (j/mm)	Dose	Médicaments	Date (j/mm)	Dose
Amoxicilline			Anti-Helminthes		
Anti-Palu.			Vaccination Rougeole		
Autres			Autres		

Traitement Spécifique

Date (j/mm)	Observations	Traitement

Sortie : Date de Sortie ..../......./.......

Guéri ☐ Abandon ☐ cause...

Décès ☐ cause.................................

Référence Vers URENI ☐ cause..

Non Réponse ☐ cause

203

Admission et à chaque visite
Les signes de danger à chaque visite

Admission et à chaque visite
Quantité ATPE à chaque visite

Décharge
Type de sortie

FICHE DE TRANSFERT

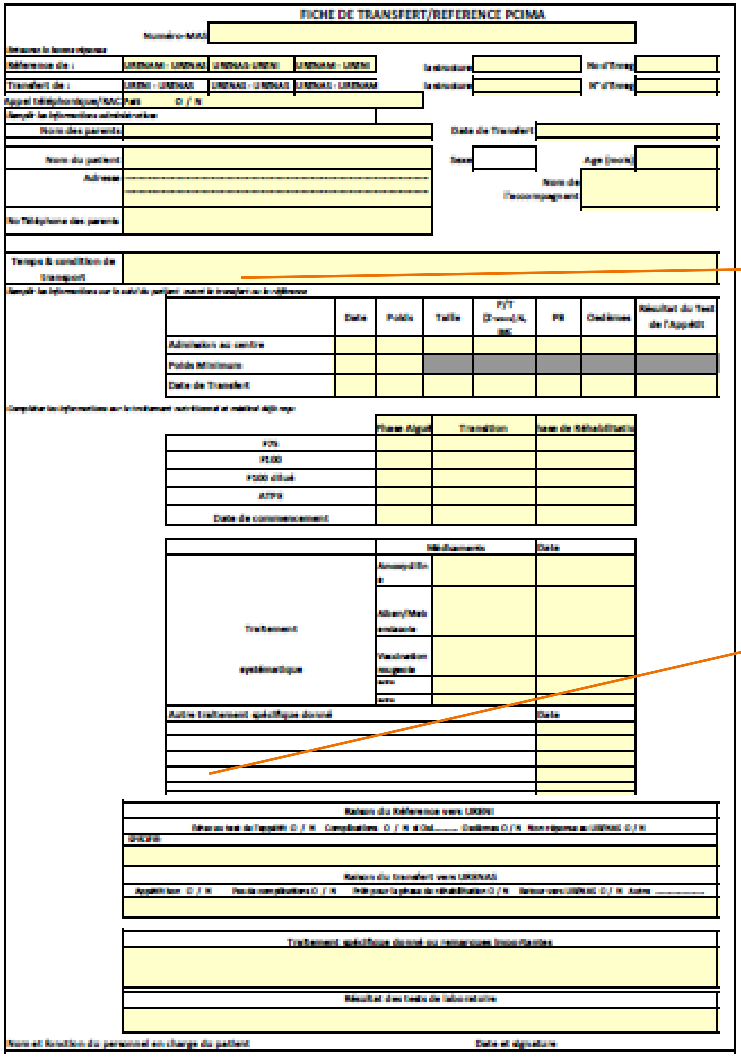

Remplir la date et mesures anthropométriques à l'admission

Remplir la raison du transfert/référence

RAPPORT MENSUEL

RAPPORT STATISTIQUE MENSUEL - URENAS/URENAM

REGION : ____________________

District: ____________________

CSCom: ____________________ Hopital: ________________ CSREF: ________________

Rapport préparé par: ________________

MOIS / ANNEE du rapport: du au

	Total début du mois			Admission				Total adm		Transfert UREN		Total général Admission = Total adm+Transfert UREN	
				Nouveau cas		Readmission							
	M	F	Total	M	F	M	F	M	F	M	F	M	F
**URENAS**													
6-59 mois													
>59 mois													
**URENAM**													
6-23 mois													
24-59 mois													
sup 59 mois													
FE/FA													
Ex MAS													

URENAS	Guéris		Décès		Abandons		Total Sorties		Référés URENI/transferts		Total sortie general		TOTAL FIN DU MOIS	
	M	F	M	F	M	F	M	F	M	F	M	F	M	F
6-59 mois														
>59 mois														
**URENAM**														
6-23 mois														
24-59 mois														
> 59 mois														
FE/FA														
Ex MAS														

cas spécifique	6-59 mois	> 59 mois	FE/FA
Nombre de tuberculeux			
Nombre de PV VIH			

Activites éducatives	Nombre
Séances CCSC réalisées	
Séances psychocognitives réalisées	
Séances de demonstration nutritionnelle réalisées	

RECOMMANDATIONS POUR L'ASC

	Quoi remplir	Quand
L'accueil	Registre	Admission
l'identification signes de danger	Registre<br>Fiche individuelle de suivi enfant MAS	Admission (semaine 1)<br>Toutes les visites
Classification de l'enfant, triage	Registre<br>Fiche individuelle de suivi enfant MAS	Admission
Traitement nutritionnel	Fiche individuelle de suivi enfant MAS<br>Fiche de suivi de stock	Admission (semaine 1)<br>Toutes les visites
Traitement systématique	Fiche individuelle de suivi enfant MAS<br>Fiche de suivi de stock	Admission (semaine 1)<br>Visite (semaine 2 et semaine 4)

RECOMMANDATIONS POUR L'ASC

	Quoi remplir	Quand
L’accueil	Registre	Admission
l’identification signes de danger	Registre<br>Fiche individuelle de suivi enfant MAS	Admission (semaine 1)<br>Toutes les visites
Classification de l’enfant, triage	Registre<br>Fiche individuelle de suivi enfant MAS	Admission
Traitement nutritionnel	Fiche individuelle de suivi enfant MAS<br>Fiche de suivi de stock	Admission (semaine 1)<br>Toutes les visites
Traitement systématique	Fiche individuelle de suivi enfant MAS<br>Fiche de suivi de stock	Admission (semaine 1)<br>Visite (semaine 2 et semaine 4)
Référencement	Fiche de référencement	A toute visite

RECOMMANDATIONS POUR L'ASC

	Quoi remplir	Quand
Référencement	Fiche de référencement	A toute visite
Sortie	Registre<br>Fiche individuelle de suivi enfant MAS	Dernière visite Visite

Insérez votre site web
