# Supplementary material for: Effectiveness and Coverage of Treatment for Severe Acute Malnutrition Delivered by Community Health Workers in the Guidimakha Region, Mauritania
Source: Children (Basel). 2021 Dec 4;8(12):1132. doi: 10.3390/children8121132 (PMC8700149; doi:10.3390/children8121132)
Supplement: Supplementary file 1 [file children-08-01132-s001.zip › Supplementary material 1.pdf]

République Islamique de Mauritanie

AGENDA DE FORMATION DES ASC SUR 21 JOURS

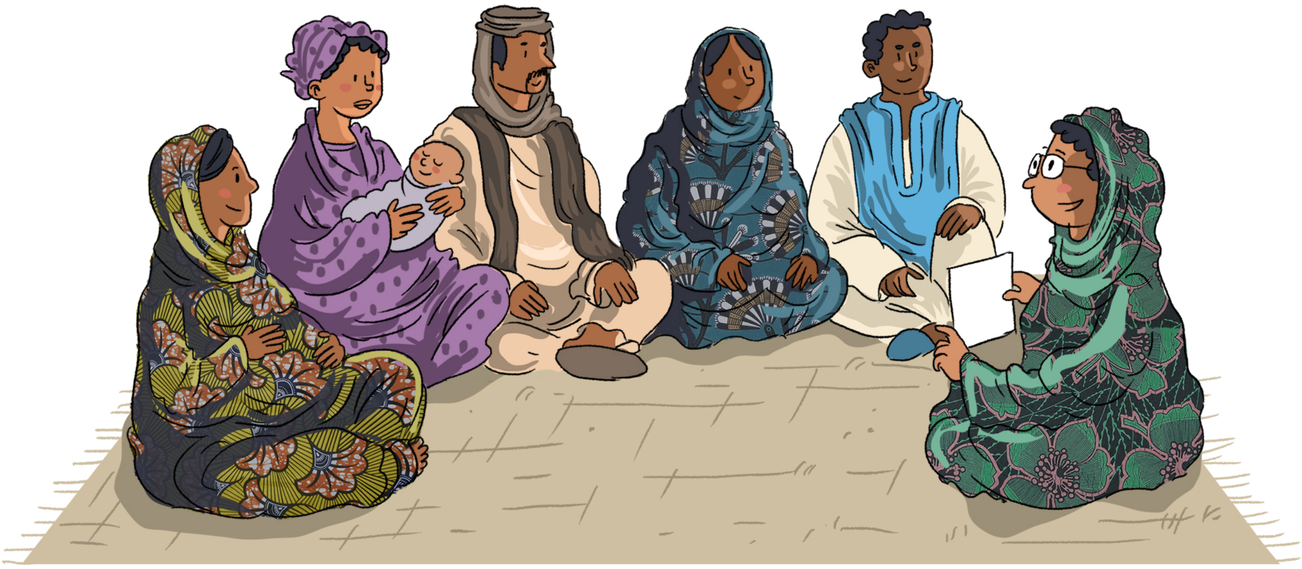

unicef

SOMMAIRE

1. Jour 1 3
2. Jour 2 4
3. Jour 3 5
4. Jour 4 6
5. Jour 5 7
6. Jour 6 8
7. Jour 7 9
8. Jour 8 10
9. Jour 9 11
10. Jour 10 12
11. Jour 11 13
12. Jour 12 14
13. Jour 13 15
14. Jour 14 16
15. Jour 15 17
16. Jour 16 18
17. Jour 17 19
18. Jour 18 20
19. Jour 19 21
20. Jour 20 22
21. Jour 21 23

01 JOUR 1

HORAIRES	ACTIVITÉS	RESPONSABLES
8H30 - 9H00	SESSION 0 : INTRODUCTION (Ouverture, Présentation des participants, Normes, calendrier, désignation rapporteurs, chef de village, time keeper.	
9H00 - 10H00	Partie I : Orientations, rôle et travail de l'ASC dans la communauté.	
10H00 - 10H30	*Pause-Café*	
10H30 - 11H30	Partie II : Séance introductive<br>Rappel du cadre de la mise en œuvre de PCIME-C<br>Organisation de PCIME-C Objectif d'apprentissage	
11H30 - 13H30	SESSION 1 : Accueil de la mère, recueil des plaintes et identification du problème	
13H30 - 14H30	*Pause-Déjeuner*	
14H30 - 16H00	SESSION 1 : Prendre les constantes : la température et le périmètre brachial (PB)	
16H00 - 16H30	*Pause-Café*	
16H30 - 17H00	Synthèse de la journée	
17H00 - 17H30	Réunion des facilitateurs	

02 JOUR 2

HORAIRES	ACTIVITÉS	RESPONSABLES
8H30 - 10H00	SESSION 1 : Prendre les constantes : la température et le péri-mètre brachial (PB)	
10H00 - 10H30	*Pause-Café*	
10H30 - 13H30	SESSION 1 : Pratique sur la prise de la T° et MUAC	
13H30 - 14H30	*Pause-Déjeuner*	
14H30 - 16H00	SESSION 2: SIGNES GENERAUX DE DANGER	
16H00 - 16H30	*Pause-Café*	
16H30 - 17H00	Synthèse de la journée	
17H00 - 17H30	Réunion des facilitateurs	

03 JOUR 3

HORAIRES	ACTIVITÉS	RESPONSABLES
8H30 - 10H00	SESSION 2 : Exercices et jeux de rôle	
10H00 - 10H30	*Pause-Café*	
10H30 - 13H30	SESSION 2 : Exercices et Jeux de rôle, pratique.	
13H30 - 14H30	*Pause-Déjeuner*	
14H30 - 16H00	SESSION 2 : Note de référence et contre référence	
16H00 - 16H30	*Pause-Café*	
16H30 - 17H00	Synthèse de la journée	
17H00 - 17H30	Réunion des facilitateurs	

04 JOUR 4

HORAIRES	ACTIVITÉS	RESPONSABLES
8H30 - 9H00	SESSION 2 : Exercices, jeux de rôle, vidéo	
9H00 - 10H00	SESSION 2 : Fiche de prise en charge des soins	
10H00 - 10H30	*Pause-Café*	
10H30 - 13H30	SESSION 3 : Fièvre / Paludisme	
13H30 - 14H30	*Pause-Déjeuner*	
14H30 - 16H00	SESSION 3 : Fièvre / Paludisme, comment faire TDR	
16H00 - 16H30	*Pause-Café*	
16H30 - 17H00	Synthèse de la journée	
17H00 - 17H30	Réunion des facilitateurs	

05 JOUR 5

HORAIRES	ACTIVITÉS	RESPONSABLES
8H30 - 10H00	SESSION 3 : Fièvre / Paludisme, comment faire TDR	
10H00 - 10H30	*Pause-Café*	
10H30 - 13H30	SESSION 4 : Toux ou rhume et difficultés respiratoires	
13H30 - 14H30	*Pause-Déjeuner*	
14H30 - 16H00	SESSION 4 : Toux ou rhume<br>Comment les évaluer et les répertorier	
16H00 - 16H30	*Pause-Café*	
16H30 - 17H00	Synthèse de la journée	
17H00 - 17H30	Réunion des facilitateurs	

06 JOUR 6

HORAIRES	ACTIVITÉS	RESPONSABLES
8H30 - 10H00	SESSION 5 : La diarrhée	
10H00 - 10H30	*Pause-Café*	
10H30 - 13H30	SESSION 5 : La diarrhée<br>Comment l'évaluer et la répertorier	
13H30 - 14H30	*Pause-Déjeuner*	
14H30 - 16H00	SESSION 5 : La diarrhée, Exercice pratique et jeux de rôles	
16H00 - 16H30	*Pause-Café*	
16H30 - 17H00	Synthèse de la journée	
17H00 - 17H30	Réunion des facilitateurs	

07 JOUR 7

HORAIRES	ACTIVITÉS	RESPONSABLES
8H30 - 10H00	SESSION 6 : La Malnutrition chez l'enfant de 6 à 59 mois, Exercice	
10H00 - 10H30	*Pause-Café*	
10H30 - 13H30	SESSION 6 : La Malnutrition<br>Le dépistage actif, exercice	
13H30 - 14H30	*Pause-Déjeuner*	
14H30 - 16H00	SESSION 7 : La vaccination, Exercice	
16H00 - 16H30	*Pause-Café*	
16H30 - 17H00	Synthèse de la journée	
17H00 - 17H30	Réunion des facilitateurs	

08 JOUR 8

HORAIRES	ACTIVITÉS	RESPONSABLES
8H30 - 10H00	SESSION 8: Traiter l'enfant et conseiller la mère : Le Paludisme	
10H00 - 10H30	*Pause-Café*	
10H30 - 13H30	SESSION 8: Traiter l'enfant et conseiller la mère : Le Paludisme	
13H30 - 14H30	*Pause-Déjeuner*	
14H30 - 16H00	SESSION 8: Traiter l'enfant et conseiller la mère : Le Paludisme, exercice pratique	
16H00 - 16H30	*Pause-Café*	
16H30 - 17H00	Synthèse de la journée	
17H00 - 17H30	Réunion des facilitateurs	

09 JOUR 9

HORAIRES	ACTIVITÉS	RESPONSABLES
8H30 - 10H00	SESSION 8: Traiter l'enfant et conseiller la mère : La toux et le rhume	
10H00 - 10H30	*Pause-Café*	
10H30 - 13H30	SESSION 8: Traiter l'enfant et conseiller la mère : La toux et le rhume	
13H30 - 14H30	*Pause-Déjeuner*	
14H30 - 16H00	SESSION 8: Traiter l'enfant et conseiller la mère : La toux et le rhume, exercice pratique	
16H00 - 16H30	*Pause-Café*	
16H30 - 17H00	Synthèse de la journée	
17H00 - 17H30	Réunion des facilitateurs	

10 JOUR 10

HORAIRES	ACTIVITÉS	RESPONSABLES
8H30 - 10H00	SESSION 8 : Traiter l'enfant et conseiller la mère : La diarrhée	
10H00 - 10H30	*Pause-Café*	
10H30 - 13H30	SESSION 8 : Traiter l'enfant et conseiller la mère : La diarrhée	
13H30 - 14H30	*Pause-Déjeuner*	
14H30 - 16H00	SESSION 8 : Traiter l'enfant et conseiller la mère : La diarrhée, exercice pratique	
16H00 - 16H30	*Pause-Café*	
16H30 - 17H00	Synthèse de la journée	
17H00 - 17H30	Réunion des facilitateurs	

11 JOUR 11

HORAIRES	ACTIVITÉS	RESPONSABLES
8H30 - 10H00	SESSION 8: Traiter l'enfant et conseiller la mère : La malnutrition aiguë sévère	
10H00 - 10H30	*Pause-Café*	
10H30 - 13H30	SESSION 8: Traiter l'enfant et conseiller la mère : La malnutrition aiguë sévère	
13H30 - 14H30	*Pause-Déjeuner*	
14H30 - 16H00	SESSION 8: Traiter l'enfant et conseiller la mère : La malnutrition aiguë sévère, exercice pratique	
16H00 - 16H30	*Pause-Café*	
16H30 - 17H00	Synthèse de la journée	
17H00 - 17H30	Réunion des facilitateurs	

12

JOUR 12

HORAIRES	ACTIVITÉS	RESPONSABLES
8H30 - 10H00	SESSION 8 : Quand retourner à l'ASC	
10H00 - 10H30	*Pause-Café*	
10H30 - 13H30	SESSION 8 : La visite de suivi<br>Le suivi des soins à domicile	
13H30 - 14H30	*Pause-Déjeuner*	
14H30 - 16H00	SESSION 8 : La visite de suivi<br>Le suivi des soins à domicile, exercice jeux de rôles	
16H00 - 16H30	*Pause-Café*	
16H30 - 17H00	Synthèse de la journée	
17H00 - 17H30	Réunion des facilitateurs	

13

JOUR 13

HORAIRES	ACTIVITÉS	RESPONSABLES
8H30 - 10H00	SESSION 8 : La visite de suivi<br>Le suivi des soins à domicile, exercice jeux de rôles	
10H00 - 10H30	*Pause-Café*	
10H30 - 13H30	SESSION 8 : La visite de suivi<br>Le suivi des soins à domicile, exercice jeux de rôles	
13H30 - 14H30	*Pause-Déjeuner*	
14H30 - 16H00	SESSION 9 : GESTION DES ACTIVITES<br>Présentation des outils de gestion	
16H00 - 16H30	*Pause-Café*	
16H30 - 17H00	Synthèse de la journée	
17H00 - 17H30	Réunion des facilitateurs	

14

JOUR 14

HORAIRES	ACTIVITÉS	RESPONSABLES
8H30 - 10H00	SESSION 9 : GESTION DES ACTIVITES<br>Présentation des outils de gestion	
10H00 - 10H30	*Pause-Café*	
10H30 - 13H30	SESSION 9 : GESTION DES ACTIVITES<br>Présentation des outils de gestion	
13H30 - 14H30	*Pause-Déjeuner*	
14H30 - 16H00	SESSION 9 : GESTION DES ACTIVITES<br>Le stockage et l'inventaire des médicaments	
16H00 - 16H30	*Pause-Café*	
16H30 - 17H00	Synthèse de la journée	
17H00 - 17H30	Réunion des facilitateurs	

15 JOUR 15

HORAIRES	ACTIVITÉS	RESPONSABLES
8H30 - 10H00	SESSION 9 : Gestion des activités<br>Le lieu de stockage	
10H00 - 10H30	*Pause-Café*	
10H30 - 13H30	SESSION 9 : Gestion des activités<br>L'inventaire physique	
13H30 - 14H30	*Pause-Déjeuner*	
14H30 - 16H00	SESSION 9 : Gestion des activités<br>La commande des médicaments	
16H00 - 16H30	*Pause-Café*	
16H30 - 17H00	Synthèse de la journée	
17H00 - 17H30	Réunion des facilitateurs	

16 JOUR 16

HORAIRES	ACTIVITÉS	RESPONSABLES
8H30 - 10H00	SESSION 9 : Gestion des activités<br>Elaboration du plan d'action de l'ASC<br>Exercices pratiques	
10H00 - 10H30	*Pause-Café*	
10H30 - 13H30	SESSION 9 : Gestion des activités<br>Elaboration du plan d'action de l'ASC<br>Exercices pratiques	
13H30 - 14H30	*Pause-Déjeuner*	
14H30 - 16H00	SESSION 9 : Gestion des activités<br>Elaboration du plan d'action de l'ASC<br>Exercices pratiques	
16H00 - 16H30	*Pause-Café*	
16H30 - 17H00	Synthèse de la journée	
17H00 - 17H30	Réunion des facilitateurs	

17 JOUR 17

HORAIRES	ACTIVITÉS	RESPONSABLES
8H30 - 10H00	SESSION 10 : Remplir les outils de gestion	
10H00 - 10H30	*Pause-Café*	
10H30 - 13H30	SESSION 10 : Remplir les outils de gestion	
13H30 - 14H30	*Pause-Déjeuner*	
14H30 - 16H00	SESSION 10 : Remplir les outils de gestion	
16H00 - 16H30	*Pause-Café*	
16H30 - 17H00	Synthèse de la journée	
17H00 - 17H30	Réunion des facilitateurs	

18 JOUR 18

HORAIRES	ACTIVITÉS	RESPONSABLES
8H30 - 10H00	La promotion des pratiques familiales essentielles	
10H00 - 10H30	*Pause-Café*	
10H30 - 13H30	La promotion des pratiques familiales essentielles / Exercice pratiques	
13H30 - 14H30	*Pause-Déjeuner*	
14H30 - 16H00	La promotion des pratiques familiales essentielles / Exercice pratiques	
16H00 - 16H30	*Pause-Café*	
16H30 - 17H00	Synthèse de la journée	
17H00 - 17H30	Réunion des facilitateurs	

19

JOUR 19

HORAIRES	ACTIVITÉS	RESPONSABLES
8H30 - 10H00	Les techniques de communication	
10H00 - 10H30	*Pause-Café*	
10H30 - 13H30	Les techniques de communication<br>Exercices pratiques et jeux de rôles	
13H30 - 14H30	*Pause-Déjeuner*	
14H30 - 16H00	Les techniques de communication<br>Exercices pratiques et jeux de rôles	
16H00 - 16H30	*Pause-Café*	
16H30 - 17H00	Synthèse de la journée	
17H00 - 17H30	Réunion des facilitateurs	

20

JOUR 20

HORAIRES	ACTIVITÉS	RESPONSABLES
8H30 - 10H00	Pratiques cliniques	
10H00 - 10H30	*Pause-Café*	
10H30 - 13H30	Pratiques cliniques	
13H30 - 14H30	*Pause-Déjeuner*	
14H30 - 16H00	Pratiques cliniques	
16H00 - 16H30	*Pause-Café*	
16H30 - 17H00	Synthèse de la journée	
17H00 - 17H30	Réunion des facilitateurs	

21 JOUR 21

HORAIRES	ACTIVITÉS	RESPONSABLES
8H30 - 10H00	Post-test et synthèse générale de la formation	
10H00 - 10H30	*Pause-Café*	
10H30-11H30	Présentation des ASC aux autorités	
11H30-12H30	Présentation des ASC aux autorités	
12H30-13H30	Clôture de la formation	
13H30 - 14H30	*Pause-Déjeuner*	
