# Supplementary material for: Effectiveness and Coverage of Treatment for Severe Acute Malnutrition Delivered by Community Health Workers in the Guidimakha Region, Mauritania
Source: Children (Basel). 2021 Dec 4;8(12):1132. doi: 10.3390/children8121132 (PMC8700149; doi:10.3390/children8121132)
Supplement: Supplementary file 1 [file children-08-01132-s001.zip › Supplementary material 2.pdf]

République Islamique de Mauritanie

Prise En Charge Intégrée des Maladies de l’Enfant par les Agents de Santé Communautaires

MANUEL DE L’ASC 2017

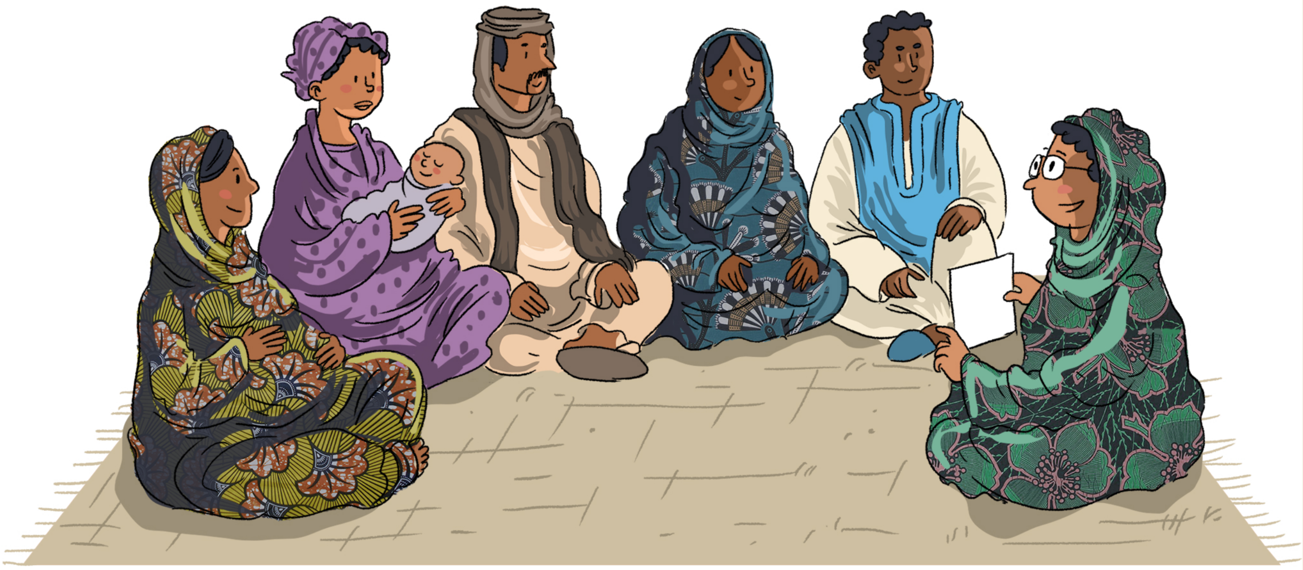

unicef

Table de matières

Contexte 5
Session 1 : Généralités sur les maladies à prendre en charge en communauté 7
Session 2 : Enregistrement du patient, recueil des plaintes et demarches des soins........ 9
Session 3 : Reconnaissance des signes de danger ou de gravite des maladies et reference des malades 11
Session 4 : Prise des constnates 14
Session 5 : Fievre / paludisme 16
Session 6 : Toux, rhume ou difficultes respiratoires 23
Session 7 : La diarrhee 26
Session 8 : La malnutrition 31
Session 9 : La vaccination 35
Session 10 : Surveillance a base communautaire 38

Abréviations

Préface

Liste des auteurs

Dr. Aly Ould Cheibany .... DSBN
Dr. Abdellahi Ould El Vally .... MASEF
Dr. Oumar Ould Sidi Mohamed .... DRAS/ASSABA
Dr. Sidi Ould Mohamed Lemine .... UNICEF
Dr. Daouda Coulibaly .... WVI
Mr. Ahmedou Ould Armiyaou .... DPCIS
Mr. Rabani Ould Ahmed Salem .... DSBN
Mr. Ahmedou Ould Mohameden .... DSBN
Mr. Aboubekrine Ould Sidi Bouna .... DLM
Mr. Aily Ould Abed .... ONG/ADCS
Mr. Mohamed Abbass Ngaida .... WVI

Liste des membres de l'atelier de validation du document

Liste des tableaux

Contexte

La redynamisation de l'approche communautaire est l'une des réformes préconisées par le PNDS 2012 – 2020 en vue du passage à l'échelle de certaines interventions à haut impact pour la survie de la mère, de l'enfant ainsi que pour la promotion de la sante en général et de la surveillance épidémiologique à base communautaire.

Dans ce cadre, le Ministère de la Santé a adopté en 2012 une stratégie de santé communautaire qui a fixé les modalités de prestation des soins au niveau communautaire par l'installation d'Unités de Santé de Base (USB) dans les petites localités éloignées des structures de santé.

Ce type de structures avait été développé par le passé et le nombre d'USB avait atteint plus de 500 au cours des années 90. Ces dernières années des USB ont été mises en place et/ou redynamisées grâce à l'appui de l'UNICEF, des ONGs (Contrepart International et World Vision) ainsi qu'aux fonds propres du gouvernement destinés à l'accélération de l'atteinte des OMD.

Les évaluations des USB conduites en 2008 et 2012 concluent que ces structures correspondent parfaitement aux besoins des communautés dans un contexte de faible densité et de dispersion des populations. Aussi elles ont un réel potentiel de contribuer à la réduction des mortalités étant donné le recours aux services encore faible et tardif et par conséquent le nombre important de décès dans la communauté.

Les principaux défis pour un tel programme de santé communautaire seraient liés à :

i) l'identification d'un paquet de tâches pertinentes, réalistes et mesurables,
ii) la disponibilité permanente des médicaments et autres intrants ;
iii) la supervision, le suivi et la coordination ;
iv) la motivation financière et non financière des ASC ; et
v) la mise en place d'un système d'information à même de permettre le monitorage des résultats et l'évaluation de l'impact des USB.

Un projet qui vise l'opérationnalisation de 500 USB dans 10 régions du pays à endémicité palustre grâce à un cofinancement de l'État, du Fonds Mondial, de l'UNICEF et d'autres partenaires a été élaboré au cours de l'année 2016. Ses activités consistent en l'identification, la formation, le suivi et la supervision des ASC conformément aux modalités prédéfinies par la Stratégie communautaire en santé.

Mais pour relever les défis suscités par les évaluations les efforts entrepris jusqu'à présent n'entraient pas dans le cadre d'un programme cohérent et coordonné. C'est pourquoi le Ministère de la santé a organisé en décembre 2015, un atelier national sur la santé communautaire, pour réaffirmer le leadership de la DSBN dans cette réforme et convenir avec l'ensemble des acteurs les principes de base et les voies et moyens d'opérationnalisation de l'approche communautaire.

Ainsi une série de recommandations avaient été adoptées y compris **le recentrage de l'action des USB autour de six thématiques** que sont :

i) iCCM ou PCIME (IRA, Diarrhées et paludisme),
ii) PCIMA (dépistage, prise en charge et orientation des enfants malnutris vers les CRENAS),
iii) Soins maternels et néonatals (orientations des femmes enceintes pour la CPN, suplémentations en Fer-Acide-Folique, Chimio prophylaxie du paludisme, Suivi des nouveau-nés avec des visites à domicile),

iv) **DOTS communautaire** (supervision de la prise de traitement chez les tuberculeux, suivi des perdus de vus et orientation des tousseurs chroniques pour le dépistage dans les CDT),

v) **Surveillance à base communautaire** (reconnaissance des signes des maladies épidémiques et leur déclaration aux autorités sanitaires) et

vi) **Promotion des pratiques familiales essentielles** à la survie de la mère, du nouveau-né et de l'enfant.

Dans le souci de renforcer la coordination du programme de santé communautaire et d'assurer la participation effective des différents acteurs dans sa conception et sa mise en œuvre, il a été également préconisé de mettre en place un comité technique de santé communautaire (CTSC).
C'est un cadre de coordination, d'échanges techniques et de recueil des informations dans le but de soumettre à la DSBN et au comité de pilotage du PNDS des recommandations fondées sur les plus récentes évolutions techniques et en fonction de la mise à jour de la situation sur le terrain.

Son objectif principal est d'orienter, de documenter et d'évaluer le programme de santé communautaire en vue de

i) le rendre le plus approprié au contexte socio-sanitaire du pays et de

ii) produire des résultats probants en termes de réduction de la morbidité et de la mortalité en particulier dans les zones non couvertes par des structures de santé conventionnelles.

Ce comité technique a veillé à ce qu'un processus de transfert de compétences aux acteurs communautaires au tour de ces six thématiques soit élaboré et mis en œuvre au cours du premier semestre de 2017 pour accompagner cette redynamisation et recentrer l'action communautaire au tour de l'accélération de la réduction de la morbi-mortalité materno-infantile en Mauritanie.

C'est ainsi que le présent Guide parmi une série d'outils a été élaboré à l'attention des formateurs des agents de santé communautaire.

Il comprend outres des sessions sur les compétences en communication que devront avoir les ASC, pour assurer leur rôle au cours des visites à domicile intégrées et celles du suivi post traitement, d'autres sessions sur **les compétences à acquérir** pour reconnaitre les signes de danger et de gravité afin de les référer immédiatement, les compétences pour prendre en charge correctement et précocement la fièvre, la pneumonie, la diarrhée, évaluer le statut vaccinal, nutritionnel de l'enfant ainsi que le suivi de sa croissance et enfin jouer pleinement son rôle dans la surveillance épidémiologique à base communautaire.

La prise en charge se fait au cours d'une **démarche systématique** qui cherche **à reconnaitre les signes de danger et de gravité** des pathologies, pour les **référer immédiatement** au niveau supérieur de la pyramide avec un **traitement pré transfert standard** et le cas échéant : **évaluer classer, traiter, conseiller la mère et suivre, selon l'algorithme de traitement** le patient qui ne présente pas de signe de danger ou de gravité.

01 SESSION 1 : GÉNÉRALITÉS SUR LES MALADIES À PRENDRE EN CHARGE EN COMMUNAUTÉ

- Liste des maladies avec leurs dénominations locales :

N°	MALADIE / AFFECTION / EVÉNEMENT	DÉFINITION DE CAS STANDARDISÉE	APPELATION LOCALE	IMAGE
1	Fièvres	Elévation de la température du corps ressentie pendant certaines maladies. Elle peut être constatée par le toucher ou mesurée par le thermomètre		
2	Pneumonies et IRA	Maladies des voies aériennes qui se manifestent par le rhume, la toux, des difficultés respiratoires ou des douleurs thoraciques		
3	La malnutrition			
4	Maladies du Nouveau né : Infections du nouveau né (ombilic, yeux, peau,..), hypothermies, faible poids à la naissance et obstructions des voix aériennes			
5	LA POLIOMYELITE / La PFA (paralysie flasque aiguë)	Tout enfant de moins de 15 ans qui rampait ou marchait et brusquement ne rampe plus ou ne marche plus		
6	LE TETANOS NEO NATAL	Tout nouveau-né capable de pleurer et de prendre le sein normalement pendant les 2 premiers jours de sa vie, et qui, entre le 3ème et 28ème jour, ne peut plus téter normalement, devient raide, a des convulsions et ou décède suite à ces signes. Tous ces signes généraux de danger du nourrisson de moins d'un mois seront référés et déclarés à l'ICP comme cas suspects de tétanos néonatal.		
7	LA ROUGEOLE	Toute personne, notamment les jeunes enfants, manifestant la fièvre et une Éruption cutanée généralisée seront référées et déclarées à l'ICP comme cas suspects de rougeole		
8	Diarrhée de l'enfant de moins de 5 ans	Tout enfant qui présente 3 selles liquide en 24 Heure avec ou sans déshydratation.		
9	Sang dans les selles	Tout enfant qui présente une diarrhée avec du sang dans les selles sera référé déclaré.		
10	LA MENINGITE	Tout individu souffrant de la fièvre et de raideur du cou.		
11	LES FIEVRES HEMORRAGIQUES VIRALES	Tout individu atteint de fièvre et présentant des saignements ou qui en est décédé.		
12	LA FIEVRE JAUNE	Tout individu souffrant de la fièvre et du jaunissement des yeux ou de la peau (plantes des pieds, ongles, paumes des mains).		
13	LE CHOLERA	Tout individu âgé de 5 ans et plus ayant des selles liquides (eau de riz) abondantes		
14	Ver de Guinée	Toute personne présentant une plaie cutanée avec sortie d'un ver blanc		
15	MAPI	Voir définition communautaire des MAPI		

- On observe souvent :
  i) négligence de la maladie,
  ii) auto médication,
  iii) recours aux guérisseurs, et
  iv) rarement recours aux structures de santé
- Les enfants atteins de ces maladies meurent de leurs complications et le plus souvent à la maison.

C'est pourquoi le rôle de l'ASC est important pour l'identification de ces maladies et la prise en charge précoce y compris la reconnaissance des signes de danger ou de gravité et leur référence immédiate vers les formations sanitaires.

Résumé :

- **Les maladies qui tuent les enfants sont : (EN LANGUES LOCALES) Causes Néonatales -IRA -Diarrhées - Fièvres/Paludisme - Malnutrition - Maladies évitables par la vaccination….**
- **Les décès surviennent en majeur partie dans les ménages**
- **Les causes de ces décès sont évitables par des simples pratiques familiales et communautaires.**
- **L'ASC joue un rôle majeur dans la prévention et la prise en charge préc**

02 SESSION 2 : ENREGISTREMENT DU PATIENT, RECUEIL DES PLAINTES ET DEMARCHES DES SOINS

1.1. Recueil des données et reconnaissance de signes de danger

DATE DU CAS	N° DU MÉNAGE	NOM	SEXE	AGE						SIGNE DE DANGER CHEZ L'ENFANT																			
				Nouveau né	1 à 11 mois	1 à 5 ans	5 à 9 ans	9 à 19 ans	20 ans et plus	Moins de 2 mois	Incapable de boire, de téter	Vomit tout ce qu'il consomme	A convulsé	Diarrhée de 14 jours ou plus	Sang dans les selles	Très affaiblie ou Inconscient	Tirage respiratoire et/ou sifflement	Désydratation sévère	Pâleur palmaire	Fièvre avec boutons sur tout le corps	raideur de la nuque	Fontanelle antérieure bombée	Œdème des 2 pieds	Urine coca cola	Amaigrissement sévère	PB < 11.5	Très affaiblie ou Inconscient	Souvent malade	Referes
1	2	3	4	5		6	7	8	9	10	11	12	13	14		15	16	17	18	19	20	21	22	23	24		25	26	27

EVALUATION CLASSIFICATION ET TRAITEMENT DE L'ENFANT													SUIVIS DES CAS À DOMICILE					Decés	AUTRES CAS PLUS DE 5 ANS											
FIÈVRE					IRA				Diarrhée				J2	J5	J8	Issue de traitement			Fiévre			Toux		Diarrhée	Femmes enceintes				Autres	
corps chaud et température	TDR +	ACT	Paracétamol	AAS	Toux	Rhume	Cotrimoxazole	Amoxycilline	Diarrhée simple	SRO	ZINC	Malnutrition modérée PB de 115 à 125				Guéri	Référé		TDR	ACT	P/AAS	< à 15 jours traitement livré	> de 5 jours Reference	traitement livré	FAF	Autre	Traitement	Référence	Plainte exprimée	traitement livré
28	29	30			31	32		33	34		35	36	38		39	40	41	42	43	44	45	46	47	48	49	50	51	52		53

1.2. Démarche des soins

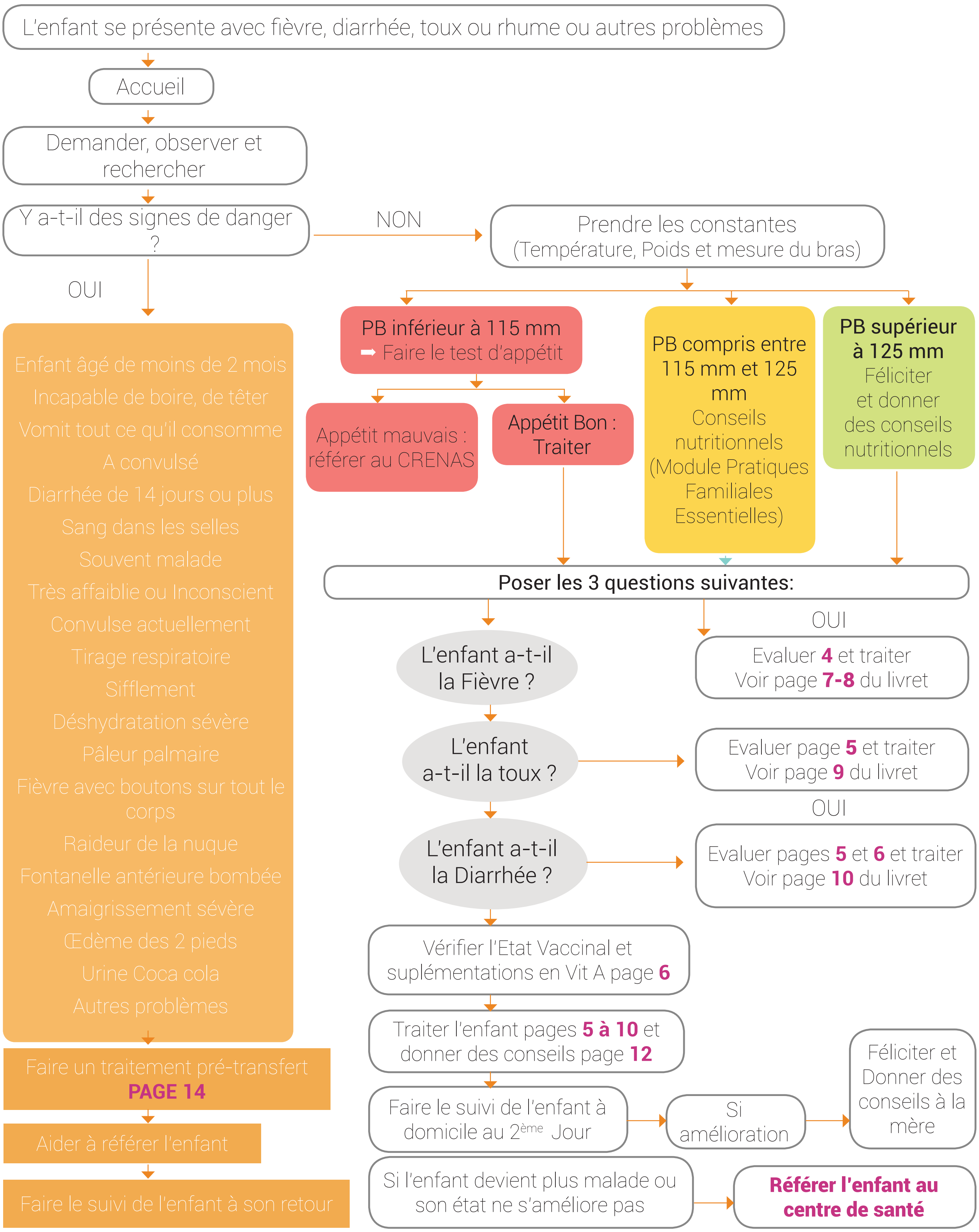

03 SESSION 3 : RECONNAISSANCE DES SIGNES DE DANGER OU DE GRAVITE DES MALADIES ET REFERENCE DES MALADES

1. LES SIGNES GENERAUX DE DANGER

N°	SIGNES	ILLUSTRATION	COMMENT RECHERCHER
1	Pâleur palmaire	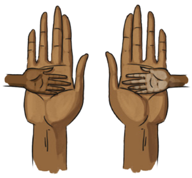	Comparer les paumes de l'ASC à celle de l'enfant : la pâleur montre une main blanche tandis qu'une bonne coloration montrera une rougeur de la paume de la main de l'enfant
2	Raideur de la nuque	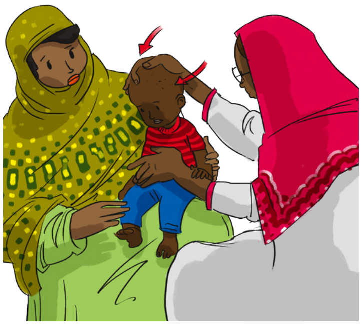	• Pendant le dialogue avec la mère au cours de l'examen, regarder si l'enfant remue et fléchit le cou sans difficulté pendant qu'il regarde autour de lui. Si l'enfant remue et plie le cou, il n'a pas de raideur.<br>• Pour rechercher passivement la raideur du cou, demander à la mère de tenir l'enfant assis sur ses cuisses ; l'ASC place une main derrière la tête de l'enfant au niveau du cou et fléchit doucement en posant l'autre main sur la poitrine de l'enfant. Si le cou se plie aisément et le « menton touche la poitrine » l'enfant n'a pas de raideur du cou ou raideur de la nuque. Si le menton ne touche pas la poitrine l'enfant a une raideur de la nuque.
3	Eruption cutanée	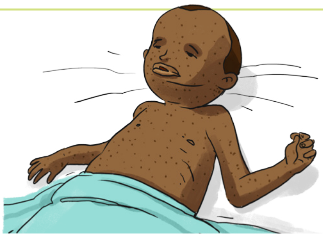	Boutons sur tout le corps. Ils sont visibles particulièrement derrière les oreilles, sur la nuque ou au visage. Ils peuvent progressivement atteindre la poitrine, le ventre et les membres. Ces boutons ne contiennent pas de liquide ni de pue. Lorsqu'ils disparaissent d'un endroit, la peau sèche et s'enlève.

2. LES SIGNES DE GRAVITE DES MALADIES

SIGNES DE DANGER ET DE GRAVITÉ	IMAGES	EQUIVALENT EN LANGUES LOCALES
Incapable de boire ou de téter et refus de s'alimenter		
Vomit tout ce qu'il consomme		
Convulsion		
Très affaibli ou Inconscient		
Respiration difficile		
Respiration rapide		
Tirage sous costal		
Cornage/stridor/geignement		
Pli cutané		
Diarrhée + sang		
Yeux enfoncés		
Diarrhée plus de 14 jour		
Agité ou irritable		
Fièvre		
Raideur de la nuque		
Eruptions cutanées généralisées		
Fièvre + saignement		
Fièvre + écoulement d'oreille		
Fièvre+gonflementdouloureuxderrièrel'oreille(mastoïdite)		
Amaigrissement visible et sévère		
Œdèmesdesdeuxpiedsquigardentlatracedelapression		
Pâleur palmaire/ conjonctivale		

DEVANT UN SEUL SIGNE DU DANGER, TERMINER VITE L'EXAMEN POUR REFERER L'ENFANT APRES L'APPLICATION DU TRAITEMENT PRE TRANSFERT

CAS À RÉFÉRER	CONSEILS À LA MÈRE POUR TOUT CAS À RÉFÉRER	ILLUSTRATION
Faciliter le transfert, ou si possible accompagner le parent et informer la communauté 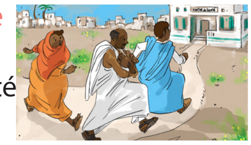 Consulter le poste de santé ou l'Unité de sante de Base la plus proche	• Si l'enfant peut téter ou boire, en route :<br>- Continuer à faire téter en cours de route (ou faire boire le lait exprimé à la tasse)<br>- Ou, si l'enfant est sevré, faire boire de l'eau sucrée (4 cuillérées à café de sucre dans 1 verre d'eau potable ou louche moyenne pour 4 carreaux de sucre)	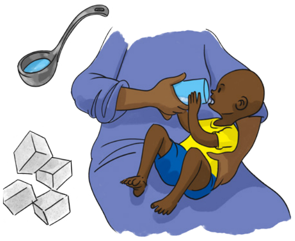
Nourrisson de moins de 2 mois 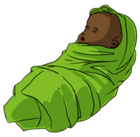	• Continuer à faire téter en cours de route (ou faire boire le lait exprimé à la tasse)<br>• Maintenir le nourrisson au chaud contre sa mère et bien couvert, pour qu'il n'ait pas froid sur le chemin du Centre de Santé	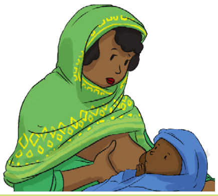
En cas de FIEVRE, 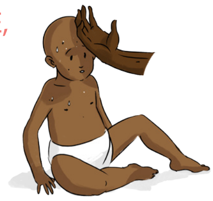	• Faire TDR, si TDR positif :<br>donner première dose d'Artesunate rectal	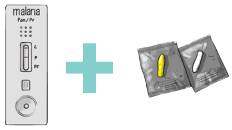
• Si FORTE FIEVRE : Température supérieure à 38,5°C	• Bain à l'eau ordinaire (tiède)<br>• ou enveloppement humide<br>+ paracétamol 500 mg (1/4 Comprimé à l'enfant de moins de 3 ans, ½ Comprimé entre 3 ans-5 ans).	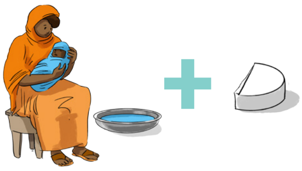
En cas de FIEVRE + RAIDEUR DE LA NUQUE ou fontanel bombée 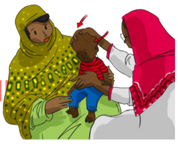	• Faire traitement de la fièvre comme précédemment et donner une première dose d'Amoxicilline selon l'âge de l'enfant	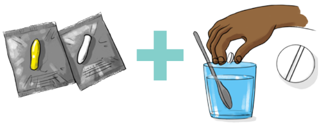
En cas de TOUX/RHUME ET PROBLEME RESPIRATOIRE 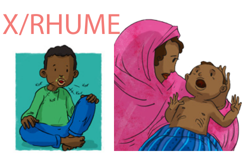	• Donner en pré-référence :<br>- l'Amoxicilline selon l'âge de l'enfant<br>• Bien couvrir l'enfant en chemin pour éviter le refroidissement.	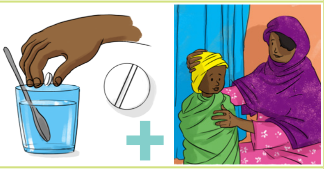
En cas de DIARRHEE : 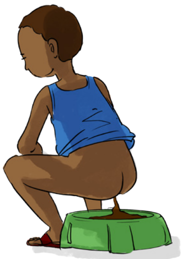	• Si l'enfant tète, continuer à le faire téter en cours de route (ou faire boire fréquemment du lait trait à la tasse),	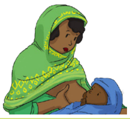
	• Et, faire boire fréquemment des gorgées de SRO avec la tasse, en cours de route (même en période d'allaitement exclusif)	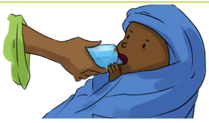
	ATTENTION. : si l'enfant a un PB inférieur 11,5 cm et/ou des œdèmes des pieds, ne pas donner du SRO ; mais conseiller de donner de l'eau sucrée	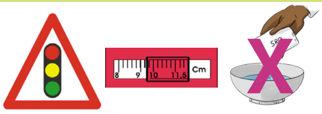
Si PB inférieur à 11,5 cm et/ou œdèmes des pieds 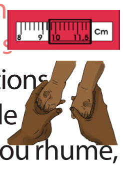 et s'il existe des complications médicales (fièvre à plus de 38° ou corps chaud, toux ou rhume, diarrhée ou autre problème)	Référer et donner le traitement de pré-transfert selon les cas.<br>Faire TDR si fièvre ou corps chaud et donner une dose d'Artesunate rectal<br>Ne pas donner de Paracétamol.	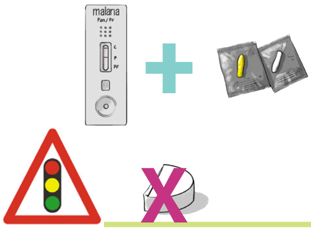

04 SESSION 4 : PRISE DES CONSTANTES

1. Mesure de la température

TÂCHES / GESTES	VÉRIFICATION
1. Préparer le matériel à portée de main (thermomètre, coton avec alcool ou de l'eau savonneuse)	
2. Expliquer au malade ou au parent ce que l'on va faire	.
3. Préparer un tampon : mouiller le coton avec de l'alcool ou de l'eau savonneuse	
4. Nettoyer le thermomètre à l'aide d'un tampon	
5. Appuyer sur le bouton « power » pendant 2 secondes, le chiffre 188.8 °C apparait en clignotant.	
6. Libérer le bouton « power » la lettre L°C	
7. Placer le bout effilé du thermomètre dans le creux de l'aisselle et attendre 5 minutes ou le bip sonore	
8. Retirer le thermomètre	
9. Lire la température sur l'écran	
10. S'il s'agit de la température axillaire, ajouter 0,5° pour obtenir la température réelle	
11. Noter la température dans le registre.	
TOTAL	

2. Mesure du périmètre brachial

On mesure le Périmètre Brachial (PB) à Mi-Hauteur du bras gauche de l'enfant pour déterminer l'état nutritionnel de l'enfant âgé de 6 mois à 5 ans.

Le PB se mesure à l'aide du mètre ruban que l'on entoure sur le bras gauche de l'enfant.

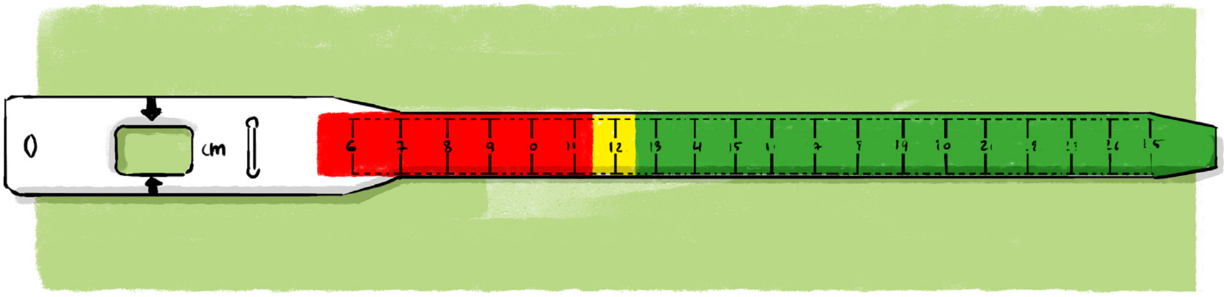

TÂCHES / GESTES	Vérification
1. Préparer le matériel à portée de main (MUAC)	
2. Expliquer au malade ou au parent ce que l'on va faire.	
3. Demander à la mère de repousser les manches de l'habit de l'enfant	
Repérage du point médian	
4. Repérer l'extrémité le sommet de l'épaule	
5. Identifier l'extrémité de l'épaule	
6. Identifier l'extrémité du coude	
7. Placer le ruban à l'extrémité de l'épaule	
8. Dérouler le ruban jusqu'à l'extrémité du coude	
9. Noter le point médian (le milieu)	
Mesure proprement dite du PB	

Ruban pour la mesure du Périmètre brachial 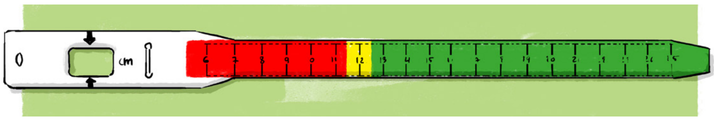

4. Repérer l'extrémité le sommet de l'épaule
5. Identifier l'extrémité de l'épaule
6. Identifier l'extrémité du coude

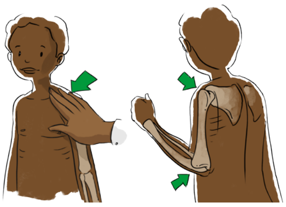

7. Placer le ruban à l'extrémité de l'épaule
8. Dérouler le ruban jusqu'à l'extrémité du coude
9. Noter le point médian

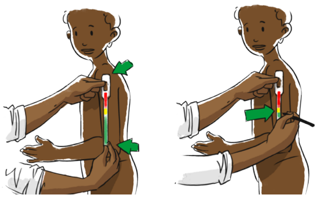

10. Maintenir le ruban pas trop lâche pas trop serré

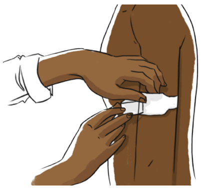

Exemple de ruban trop serré

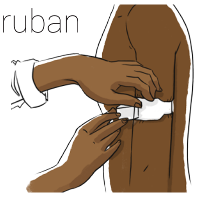

Exemple de ruban trop lâche

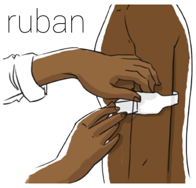

11. Faire la lecure (Position correcte pour mesurer le périmètre brachial)
12. Noter le PB dans le carnet et dans le registre

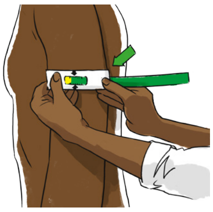

05 SESSION 5 : FIEVRE / PALUDISME

Introduction/ Généralité

La fièvre du paludisme est une maladie grave et mortelle. Elle est surtout dangereuse chez les femmes et les petits enfants.

Elle est causée exclusivement par le moustique et en particulier l'anophèle.'

C'est une maladie facile à prévenir lorsque les mesures conseils donnés par les agents de santé sont parfaitement respectés.

Si le diagnostic est fait rapidement et correctement Chez les personnes atteintes de fièvres, les patients guérissent facilement.

Toute personne gravement malade doit être référée en urgence

- **Si le paludisme n'est pas traité dans les 24 heures, le paludisme peut présenter des complications sévères souvent mortelles ;**
- **Le paludisme est une maladie évitable si on utilise correctement et continuellement les mesures de prévention (MILDA, TPI, Hygiène environnementale);**
- **Les personnes venant de régions exemptes de paludisme sont très vulnérables à la maladie lorsqu'ils sont infectés.**

Mesures pour prévenir l'aggravation :

Devant toute fièvre il faut faire le test rapide du plaudime TDR

Devant tous cas positif au TdR, commencer aussitôt le traitement

DEFINITION DE CAS

PALUDISME SIMPLE = TOUT ENFANT OU ADULTE MALADE AYANT LA FIEVRE

(= TEMPERATURE 38°C OU PLUS, CORPS CHAUD AU TOUCHER OU ANTECEDENTS DE FIEVRE DANS LES 2 JOURS PRECEDENTS) + TDR POSITIF SANS SIGNE DE DANGER.

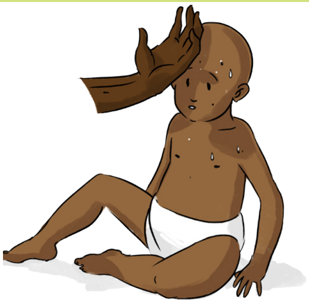

Demander

- Depuis combien de temps l'enfant a-t-il la fièvre ?
- Si plus de 7 jours, la fièvre est-elle présente tous les jours ?
- L'enfant dort-il sous moustiquaire ?
- Si oui, la moustiquaire est-elle imprégnée d'insecticide ?

PRATIQUE DU TEST RAPIDE DE DEPISTAGE

Définition :

Le test de diagnostic rapide du paludisme (TDR): est une des méthodes qui révèle la présence du parasite du paludisme à partir d'un prélèvement de sang au bout du doigt.

A qui doit-on faire le test ?

Pour les enfants de 2 mois à 5 ans et autres personnes présentant une fièvre, il est nécessaire de faire le TDR avant d'administrer le traitement, à cause du risque très élevé de survenue des signes de gravité.

a- Conservation / stockage :

- Température de conservation du TDR : Conserver entre + 4 et 40° C (Garder le TdR au frais tant que possible – à l'abri du soleil et de la chaleur)
- Conserver les tests dans leur boîte dans un lieu propre et assez sec que possible
- Garder hors de la portée des enfants

b- Composition du kit / Matériel indispensable:

Le kit de TDR est composé de 3 éléments :

- La cassette du test où le test sera effectué
- Une petite pipette pour le prélèvement de sang
- Un desséchant dont le rôle est d'absorber l'humidité

Le test est livré avec des lancettes et des tampons imbibés d'alcool.

Prévoir aussi des gants, une montre en bon état, un crayon, un marqueur fin, un cahier/registre et une boîte de sécurité pour les lancettes.

Ramener l'emballage ou le kit à la température ambiante pendant 30 minutes s'il était conservé entre +4 et 8°C.

TECHNIQUES DE LA PRATIQUE DU TDR

TÂCHES / GESTES
Apporter le matériel à portéede la main (le kit, la pipette capillaire, le lancette, les gants, le tampon, le diluant, la boîte de sécurité, le bic, la montre….) 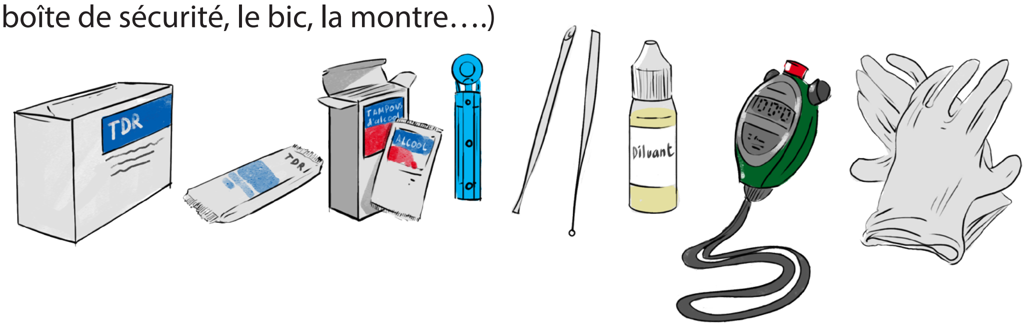
Expliquer au malade ou au parent ce que l'on va faire.
Se laver les mains et sécher à l'air libre ou avec une serviette propore 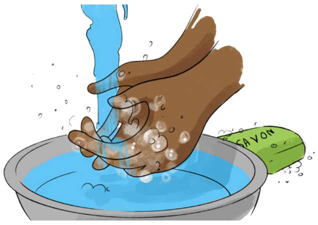
Vérifier la date d'expiration sur l'emballage du test. Voir la couleur bleu[illegible] 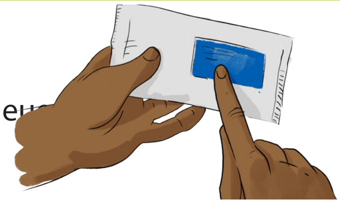
Porter les gants 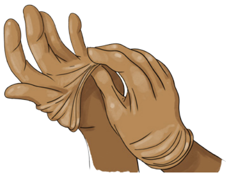
Ouvrir l'emballage et faire sortir le test 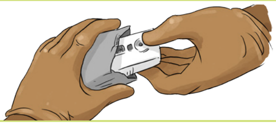
Ecrire le nom du patient sur le test et la pipette capillaire 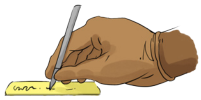
Ouvrir le sachet imbibé d'alcool. Tenir le 4ème doigt de la main gauche du patient.<br>Nettoyer le doigt avec le sachet d'alcool. Laisser le doigt devenir sec avant de piq[illegible] 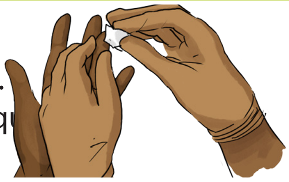
Ouvrir le lancette. Piquer le doigt du patient afin d'avoir une goutte de sang.<br>Ne laisser pas la pointe du lancette toucher autre chose avant de piquer le doigt du patient. 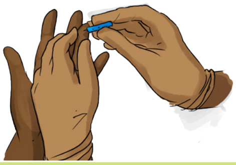
Jeter la lancette dans la boite de sécurité immédiatement après avoir piqué le doigt du patient 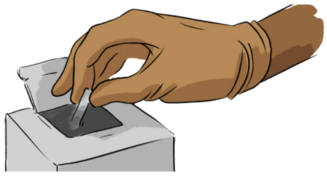

TÂCHES / GESTES
Utiliser le tube ou gobelet renversé pour collecter la goutte de sang en appliquer légèrement l'embout sur la goutte de sang, le sang sera absorbé automatiquement 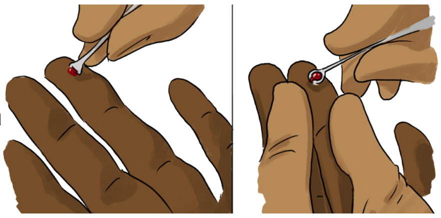
Mettre immédiatement le sang prélevé par la pipette dans le puits "rc de la cassette. En touchant le fond. Jeter la pipette dans la boîte de sé 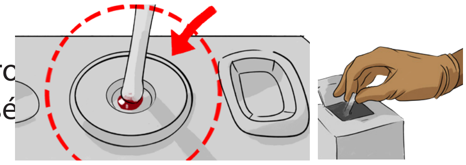
Placer un tampon de coton à l'emplacement de la piqûre pour arrêter le saignemen 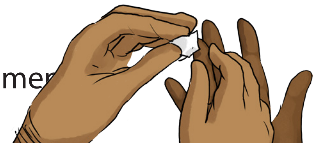
Mettre quatre (4) gouttes du diluant dans le puits "carré". A la première utilisati tourner le bouchon pour percer le flacon et permettre au diluant de couler. 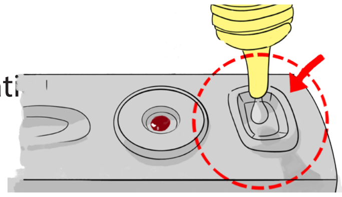
Déposer la cassette sur une surface horizontale pour permettre au sang de migrer tout au long de la zone du test
Lire le résultat exactement 15 minutes après avoir ajouté le diluant. 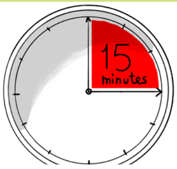
Regarder dans les sections ou fenêtres "C" et "Pf" de la cassette pour rechercher la présence ou l'absence de bande(s) de couleur rc 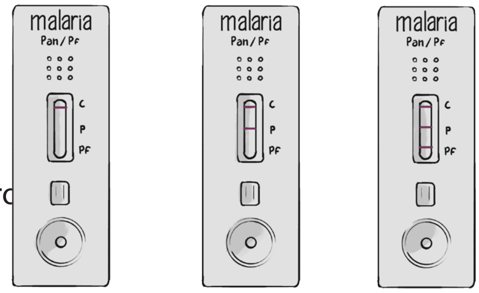 TEST NEGATIF TEST POSITIF

c- Mesures de sécurité:

- Le sang est un produit contaminant car il peut transmettre plusieurs maladies. Ainsi si le sang d'une personne infectée entre en contact avec une autre personne, il y a risque pour cette dernière d'attraper des maladies en particulier le VIH/SIDA et l'hépatite virale.
- Pour cette raison toute personne travaillant au contact du sang, doit prendre beaucoup de précaution pour ne pas être piqué et ne pas exposer les autres personnes aux objets souillés de sang.

Il faut alors :

- Utiliser les gants pendant la réalisation du test
- Jeter les lancettes utilisées dans les boites de sécurités
- Bien gérer les tampons imbibés utilisés (confère gestion des déchets)

- S'assurer que le patient ou l'enfant piqué ne saigne plus
- Toutefois si vous vous piquez ou que vous piquez accidentellement quelqu'un avec une lancette souillée, il faut dans les 6 heures suivantes avertir le responsable du poste de santé de rattachement.

d- Gestion des déchets

- L'emballage, la petite pipette, le desséchant et le tampon doivent être brûlés par l'ASC.
- Les cassettes des tests sont conservées par l'ASC pour le contrôle du responsable du poste de santé
- les lancettes (vaccinostyles) sont stockées dans les boites de sécurités ou bouteilles vides d'eau minérale et ramenées remplies au ¾ au responsable du poste de santé pour incinération

e- Les 3 niveaux de contrôle dans la réalisation du TDR :

- 1er niveau de contrôle: (avant l'ouverture du kit)
  - Regarder la date de péremption au verso du kit. Si cette date est dépassée, ne pas utiliser ce kit. Prendre un autre kit non périmé.
  - Vérifier si l'emballage du kit n'est pas endommagé. Si l'emballage est endommagé, ne pas utiliser le kit mais prendre un autre.
- 2ème niveau de contrôle : (dès l'ouverture du kit)
  - Ouvrir le kit du test juste avant usage en déchirant l'emballage : Vérifier par rapport à la lumière (soleil ou ampoule électrique) que le dessicant a une couleur bleuâtre. Si non utiliser un autre test.
- 3ème niveau de contrôle : (au moment de la lecture)
  - Pas de Trait de Contrôle dans la fenêtre "C." Le Trait du Test est présent dans la Fenêtre "Pf" mais pas celui du Contrôle.
  - Pas de trait ni dans la zone (ou fenêtre) du test "Pf" ni dans celle du contrôle "C."

  Dans les deux cas reprendre le Test avec un nouveau TDR

TRAITEMENT DU PALUDISME SIMPLE

**ARTÉSUNATE (AS) – AMODIAQUINE (AQ)**		
Administrer une (1) prise unique par jour pendant 3 jours en fonction du poids ou de l'âge de l'enfant		
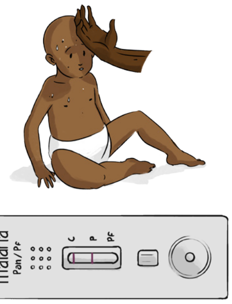	AS 25 mg AQ 67,5 mg<br>Blister de 3 cp	AS 50 mg AQ 135 mg<br>Blister de 3 cp
	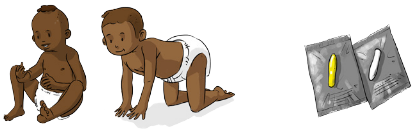 2 mois à 12 mois<br>4,5 Kg à 9 Kg	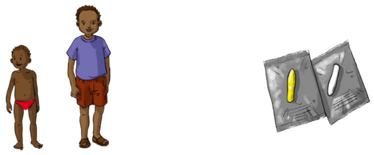 12 mois à 5 ans<br>9 Kg à moins de 19 Kg
1er jour	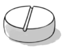	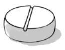
2ème jour	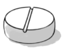	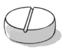
3ème jour	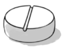	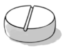

Et Administrer du paracétamol 500 mg pour une fièvre (T° supérieure à 38,5°C):

Age / Poids	Jour 1				Jour 2			
	Matin	Midi	Après-midi	Soir	Matin	Midi	Après-midi	Soir
4 kg à 14 kg (2 mois à 3 ans)	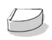		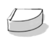		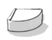		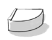	
14 kg à moins de 18 kg (3 ans à 5 ans)	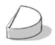		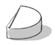		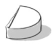		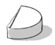	

Conseils

Les 3 règles de traitement (CAR)

1. **Continuer l'allaitement, donner des conseils nutritionnels**
2. **Augmenter les liquides à boire (ou le lait maternel en cas d'allaitement exclusif)**
3. **Reconnaître quand revenir immédiatement (c'est-à-dire l'enfant devient plus malade)**

• Conseiller à la mère de retourner immédiatement si l'enfant présente l'un de ces signes:	
Léthargique ou inconscient<br>Convulse	✓ Incapable de boire ou de téter<br>✓ Incapable de manger<br>✓ Devient plus malade<br>✓ Développe une fièvre

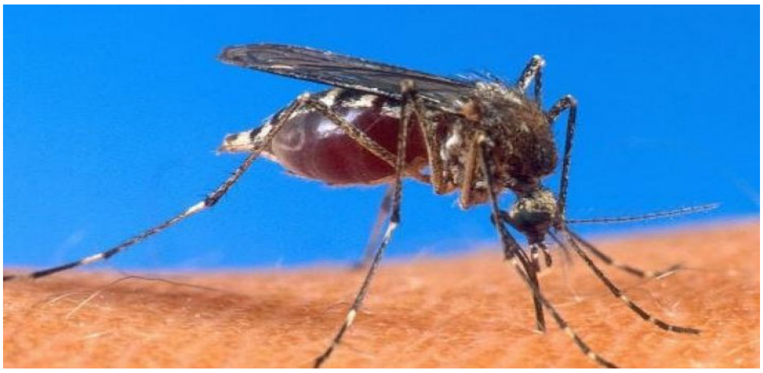

Moustique en action : quand tu dors, il mange

Evitez le contact avec les moustiques

- Dormir sous une moustiquaire imprégnée;
- Se couvrir la peau en portant des pantalons et des manches longues une fois la nuit tombée ;
- Évitez de sortir de vous exposer la nuit sauf situation obligatoire.

Pour prévenir le paludisme dans l'environnement du domicile ; Eviter les flaques d'eau souvent gites larvaire des Moustiques en les asséchant le plus rapidement possible avant 12 heures ; les ustensiles et tout contenant négligé peuvent aussi servir de gites.

La moustiquaire imprégnée reste le moyen le plus efficace pour éviter le contact avec les moustiques et prévenir le paludisme. Elle chasse et/ou tue les moustiques sans avoir d'effets néfastes sur la santé des enfants et des adultes. Elle est relativement disponible et son utilisation est très facile. Toutefois, elle doit être utilisée continuellement dès la tombée du soleil et ne doit pas être exposée au soleil ni lavée.

Remarque : Le paludisme est particulièrement dangereux chez la femme enceinte, c'est pourquoi, elle doit rester tous les soirs sous MILDA et utiliser la chimio prophylaxie prescrite pendant les CPN (au 5ème mois, 7ème et 9ème mois).

Pendant la saison des pluies (haute transmission). Initier des actions collectives dans les villages pour **Lutter contre les moustiques**

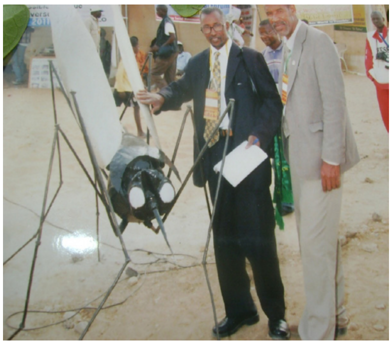

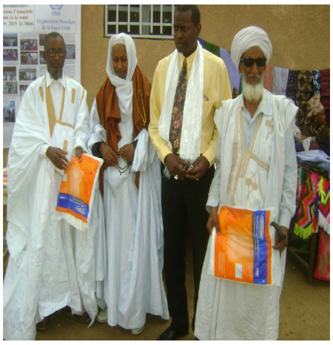

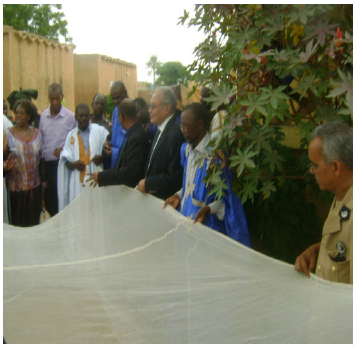

Tous les acteurs : autorités administratives, religieuses, sanitaires, communales, les leaders communautaires, les jeunes et les autres doivent être mobilisés pour mener les actions qui réduisent les moustiques dans l'environnement et assurent l'utilisation systématique des MILDA ne se reste que part les enfants et les femmes enceintes.

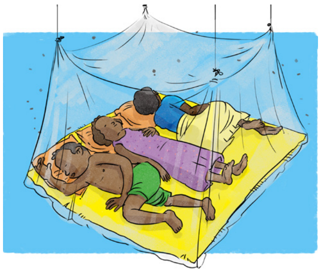

Visite à domicile pour le suivi post traitement :

L'objectif de la visite à domicile est de vérifier s'il n'y'a pas d'apparition de signes de gravité ou de danger chez les malades mis sous traitement. Si de tels signes surviennent référer le malade immédiatement.

Les malades chez qui le TDR était négatif.

Si le TDR était négatif revoir le patient dans 24 heures et agir en conséquence ;

Si l'évolution était favorable répéter la visite à J2, J5 et J8.

06 SESSION 6 : TOUX, RHUME OU DIFFICULTES RESPIRATOIRES

5.1. DEFINITION

La toux est un signe fréquent chez les enfants de moins de 5 ans. Elle est souvent causée par une maladie de l'appareil respiratoire. Il peut s'agir de toux simple ou de pneumonie ou d'une infection grave (respiration difficile)

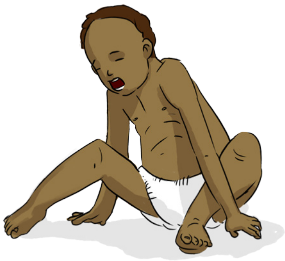

La respiration difficile : On reconnaît un enfant qui respire difficilement devant les signes suivants :

- Soit que la base (ou le sommet) de son thorax s'enfonce quand il respire (tirage sous-costal)
- Soit que l'enfant émet un bruit aigu ou rauque ou un sifflement anormal pendant qu'il respire
- Soit que les ailes du nez de l'enfant sont en train de battre pendant que l'enfant respire

DIAGNOSTIC	ILLUSTRATION
Pneumonie grave : la pneumonie grave est définie comme une toux et/ou respiration difficile avec un tirage sous costal, un bruit anormal ou sifflement	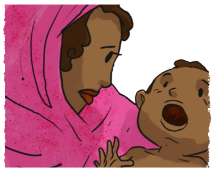 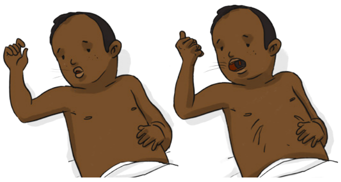
Pneumonie : la pneumonie est définie comme une toux avec une respiration rapide.	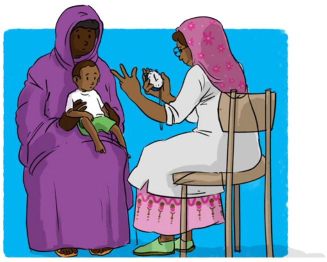

4.2. COMMENT EVALUER LA TOUX OU LA DIFFICULTE RESPIRATOIRE ?

1. Demander à la mère depuis combien de jours l'enfant tousse et/ou a de la difficulté à respirer
   Utiliser des mots décrivant la toux ou rhume que la mère peut comprendre.
   Laisser à la mère le temps de répondre. Elle peut essayer de se souvenir du nombre exact de jours.

2. Compter les mouvements respiratoires pendant une minute :

   L'enfant doit être calme et silencieux pour ne pas perturber l'observation.
   - Soulever son habit
   - Observer les mouvements respiratoires sur la poitrine ou l'abdomen de l'enfant.
   - Utiliser le chronomètre (minuteur) ou une montre avec trotteuse.

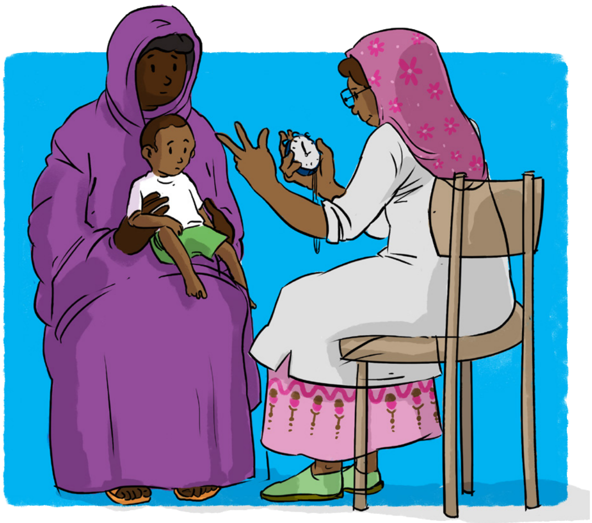

SIGNES	CLASSIFICATION
1. Rechercher tirage sous costal : Tirage ou bruit anormal oui......... non.........	PNEUMONIE GRAVE
2. Compter les mouvements respiratoires par minute............ mouvements respiratoires par minute.<br>- 2 mois à moins de 12 mois : 50 mouvements respiratoires et plus<br>- 12 mois à 59 mois : 40 mouvements respiratoires et plus<br>Respiration Rapide oui.......... non..........	PNEUMONIE
3. Absence de signe de gravité<br>• Pas de tirage sous costal ou bruit anormal<br>• Pas de respiration rapide	TOUX OU RHUME

Rappel : si l'enfant a un tirage sous costal, un bruit anormal pendant la respiration, a une respiration rapide, ou s'il tousse depuis plus de 14 jours, il doit être référé.

Traiter la pneumonie :

Administrer l'Amoxicilline dispersible en première intention

Age / Poids	AMOXICILLINE Comprimé dispersible 250 mg<br>Administrer 2 fois par jour pendant 5 jours	
	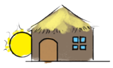<br>Matin	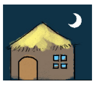<br>Soir
6 kg à 9 kg<br>(2 mois à 12 mois)	1 comprimé 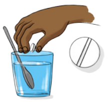	1 comprimé 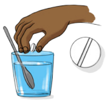
14 kg à moins de 18 kg<br>(3 ans à 5 ans)	2 comprimés 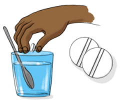	2 comprimés 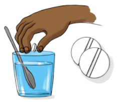

Age / Poids	COTRIMOXAZOLE (triméthoprime + sulphaméthoxazole)<br>Donner deux fois par jour pendant 5 jours	
	COMPRIMÉ ADULTE<br>80 mg triméthoprime<br>+ 400 mg sulphaméthoxazole	COMPRIMÉ ENFANT<br>20 mg triméthoprime<br>+100 mg sulphaméthoxazole
de 2 mois à 12 mois<br>(4 - <10 kg)	¼ comprimé 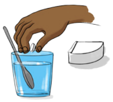	1 comprimé 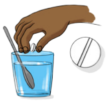
de 12 mois à 5 ans<br>(10 - 19 kg)	½ comprimés 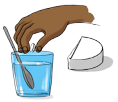	2 comprimés 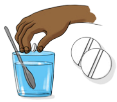

Les 3 règles de traitement (CAR)

1. Continuer l'allaitement, donner des conseils nutritionnels

2. Augmenter les liquides à boire (ou le lait maternel en cas d'allaitement exclusif)

3. Reconnaître quand revenir immédiatement (c'est-à-dire l'enfant devient plus malade)

Autres conseils sur l'hygiène et le lavage des mains aux moments opportuns (voir ci-dessous)

• Conseiller à la mère de retourner immédiatement si l'enfant présente l'un de ces signes:	
Tout enfant malade	✓ Incapable de boire ou de téter<br>✓ Incapable de manger<br>✓ Devient plus malade<br>✓ Développe une fièvre
Si l'enfant a:<br>TOUX OU RHUME, aussi retourner si:	✓ Respiration rapide<br>✓ Difficultés respiratoires

Suivre à domicile J2, J5 et J8 en recherchant à chaque fois les signes de danger pour référer immédiatement

07 SESSION 7 : LA DIARRHEE

7.1. Définitions :

On désigne par diarrhée chez les enfants moins de 5 ans la survenue d'au moins 3 selles liquides au cours des dernières 24 heures, avec ou sans signes de déshydratation

- Un enfant est atteint de la diarrhée lorsqu'il a des selles liquides 3 fois ou plus par jour
- Diarrhée sanglante ou dysenterie : le ventre fait très mal, les selles sont accompagnées de sang.
- Diarrhée persistante : la diarrhée dure 14 jours ou plus.

Les facteurs favorisant la diarrhée :

- Mains sales,
- Consommation d'aliments laissés ouverts aux mouches et à la poussière
- Les légumes et fruits mal lavés,
- Consommation de l'eau sale issue des mares et des puits mal aménagés et non traitées.

La diarrhée provoque la déshydratation c'est à dire la perte de liquides pouvant entraîner la mort. Elle peut également entraîner la malnutrition si elle dure longtemps.

7.2. Evaluer et classer la diarrhée :

Utiliser le livret pour rechercher les signes de gravité de la diarrhée

L'ENFANT A-T-IL LA DIARRHEE actuellement ? Oui___ Non___<br>RECHERCHER SYSTEMATIQUEMENT L'UN DES SIGNES DE DESHYDRATATION SUIVANTS, LA PRESENCE OU NON DE SANG ET APPRECIER LA DUREE DE LA DIARRHEE POUR REFERER IMMEDIATEMENT LES CAS GRAVES			
• Depuis combien de temps ? _____ jours<br>Inférieur à 14 jours<br>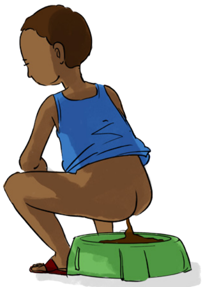<br>•Ses selles contiennent-elles du sang ? Oui ___ Non___<br>Supérieur ou égal à 14 jours<br>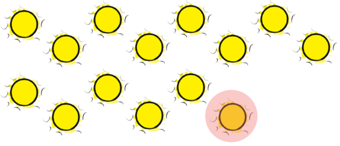	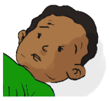 >Très fatigué ou inconscient, Incapable de boire, Yeux enfoncés<br>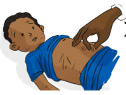 >Pli cutané très lent ? (plus de 2 secondes) Oui___Non___<br>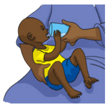 - Offrir à boire à l'enfant :<br>- Boit-il avec avidité, est-il assoiffé ? Oui___Non___<br>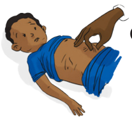 - Vérifier le pli cutané : Pli cutané s'efface lentement ? (moins de 2 secondes) Oui ___ Non___	Déshydratation	REFERER<br>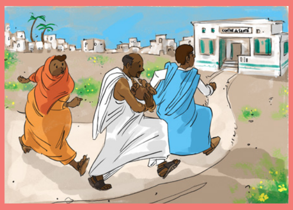
	> Diarrhée de 14 jours ou plus<br>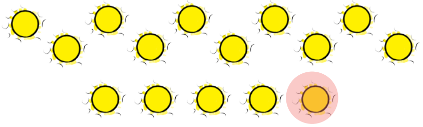	Diarrhée persistante	REFERER<br>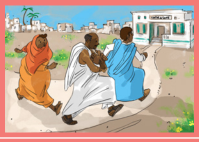
	> Sang dans les selles<br>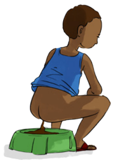	Dysenterie	REFERER<br>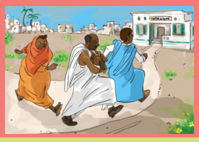
SI ABSENCE DES SIGNES PRECEDENTS DE GRAVITE, CLASSER COMME DIARRHEE SIMPLE, TRAITER AVEC SRO+ZINC ET CONSEILLER LA MERE		Diarrhée simple sans déshydratation	TRAITER (SRO et Zinc) Poursuivre l'evaluation

Référer immédiatement les cas présentant des signes de déshydratation, ayant du sang dans les selles et ceux ayant la diarrhée depuis 14 jours et plus

7.3. Traiter la diarrhée :

Préparer et administrer la SRO et le Zinc et poursuivre l'allaitement et/ou l'alimentation

Sachet SRO

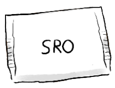

Tasse 1 litre

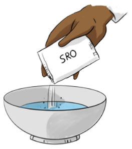

Tasse 1 litre

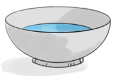

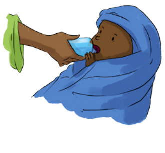

+

Zinc cp

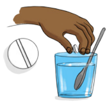

+

Poursuite allaitement

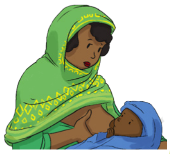

Quelle quantité de SRO faut-il donner à l'enfant après chaque selle ?

Age de l'enfant	Quantité de SRO à donner	
Jusqu'à 2 ans	½ tasse (50 à 100 ml)	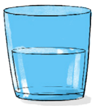
2 ans et plus	1 tasse (100 à 200 ml)	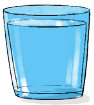

Autres liquides recommandés en cas de diarrhée : eau potable, eau de riz, potage, lait de coco, lait de soja, eau de courge

Prise de Zinc comprimés pendant 10 jours sans arrêt

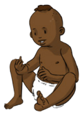 2 à 6 Mois	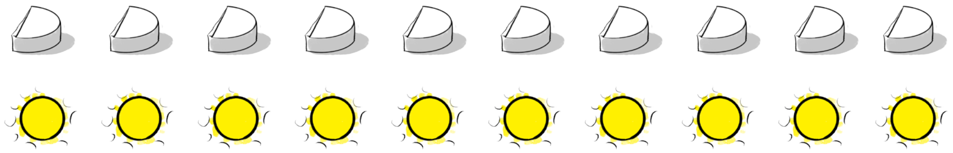
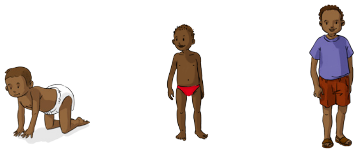 6 à 59 Mois	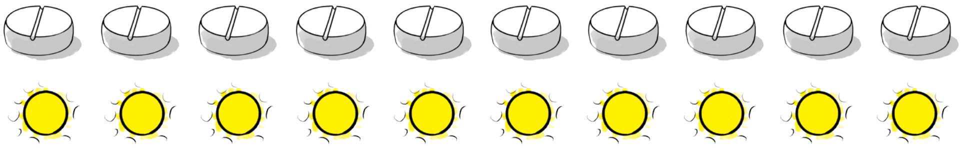

Si absence de sachets SRO, apprendre à la mère comment préparer une solution à domicile (SSS)

7.4. DEMONSTRATION DE LA PREPARATION DE LA SRO

Matériel :
- Sachet de SRO
- Bol
- Petit gobelet
- Bouteille d'un litre
- Cuillère
- Verre à thé

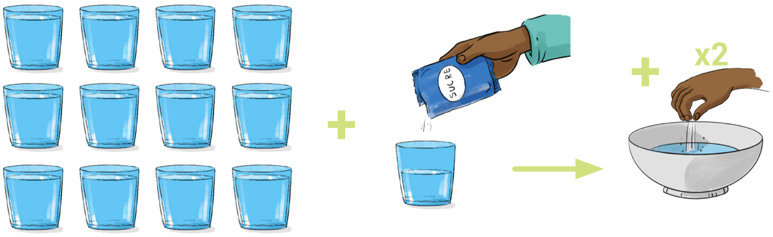

12 verres à thé d'eau propre traitée ou potable

Demi verre à thé de Sucre

2 pincées entre trois doigts de sel râpé.

7.5. CONSEILS POUR LA MÈRE

a.Les 4 règles de traitement de la diarrhée

1. Continuer l'allaitement, donner des conseils nutritionnels
2. Augmenter les liquides à boire (SRO+ le lait maternel en cas d'allaitement exclusif)
3. Donner du Zinc : un comprime par jour pendant 10 Jours
4. Reconnaître quand revenir immédiatement (c'est-à-dire l'enfant devient plus malade)

• Conseiller à la mère de retourner immédiatement si l'enfant présente l'un de ces signes:	
Tout enfant malade	✓ Incapable de boire ou de téter<br>✓ Incapable de manger<br>✓ Devient plus malade<br>✓ Développe une fièvre
L'enfant ayant la diarrhée :<br>il faudra revenir immédiatement	✓ Si sang dans les selles<br>✓ Si la diarrhée s'aggrave (fréquence et signes de déshydratation)

Autres conseils sur l'hygiène et le lavage des mains aux moments opportuns (voir ci-dessous)

b. CONSEILLER DE CONTINUER LE TRAITEMENT DE L'ENFANT (Zinc et SRO)

- Demander au parent de rappeler comment il a administré les médicaments
- Si le parent a bien administré les médicaments, FELICITER ET ENCOURAGER A CONTINUER
- Si le parent a mal administré les médicaments, DEMONTRER COMMENT ADMINISTRER, puis demander lui de répéter et d'administrer une dose en votre présence. Vérifier sa compréhension
- Une solution SRO préparée ne doit plus etre utilisée au delà de 24heures après sa préparation.

c. AUTRES CONSEILS EN CAS DE DIARRHEE

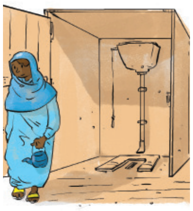

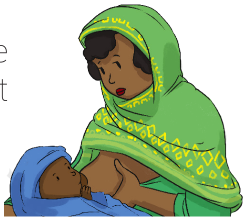

-Continuer l'allaitement maternel à volonté, donner SRO après chaque selle liquide à raison de : un demi verre à thé pour les moins d'un an et un verre à thé pour les plus d'un an

-Utiliser les latrines familiales et les maintenir propres et les désinfecter

-Laver les mains après la sortie des toilettes, après changement de couches des bébés et soins des malades, avant la préparation des repas et avant de manger

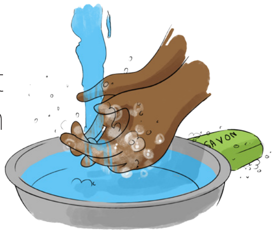

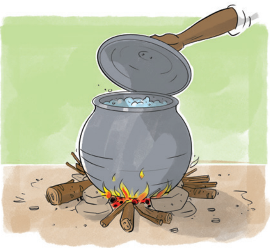

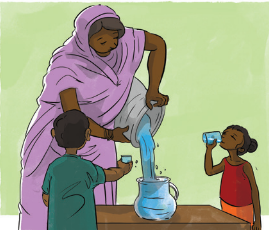

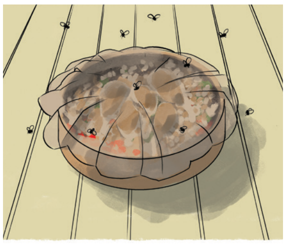

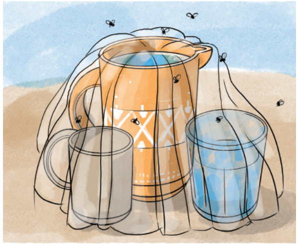

-Bouillir l'eau pour tuer les microbes, la filtrer et la garder dans des récipients propres et protéger l'eau et les aliments contre les insectes et mouches.

Traitement et désinfection de l'eau turbide

Comment rendre l'eau clair:
L'eau d'un puit ouvert ou d'un marigot n'est pas potable
même si elle semble être propore. Cette eau est contaminée par:

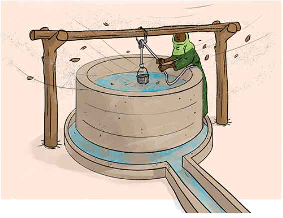

La poussière

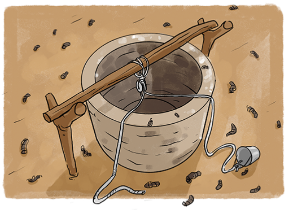

Les excréments des animaux

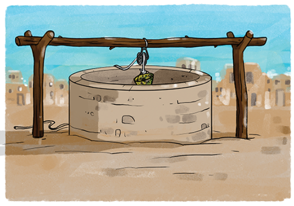

Une puisette sale

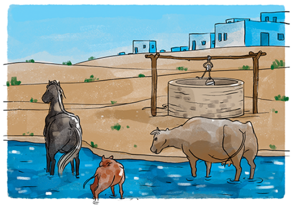

Des animaux

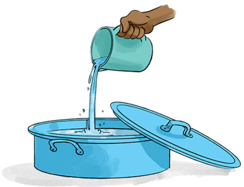

Mettre l'eau trouble dans une marmite

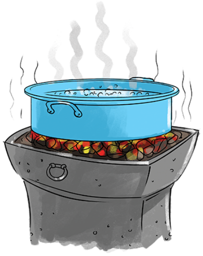

Faire bouillir l'eau

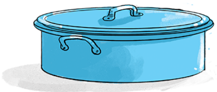

Laisser refroidir

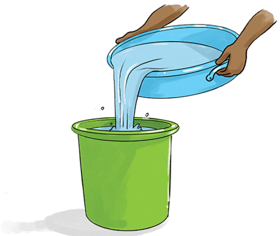

Elle devient potable

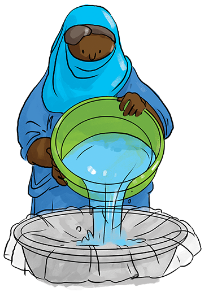

Filtre avec tissu

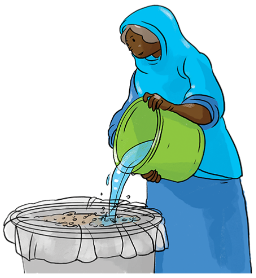

Filtre avec sable

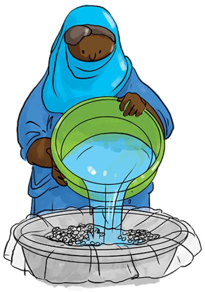

Filtre avec gravillon

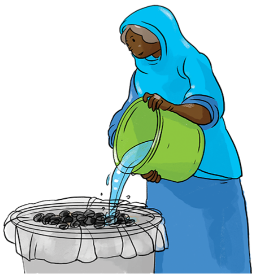

Filtre avec charbon

En javélisant :

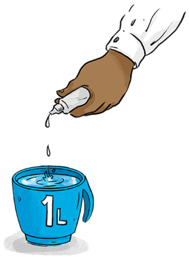

Pour 1 litre d'eau
2ou 3 gouttes

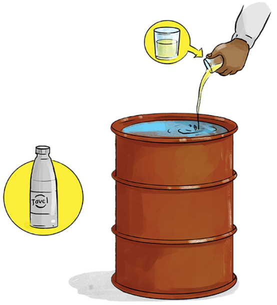

Pour une barrique
de 200 litres
un demi verre à thé

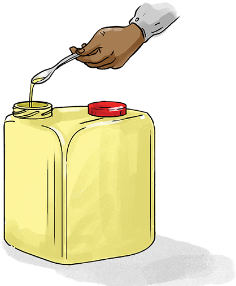

Pour un bidon
de 20 litres

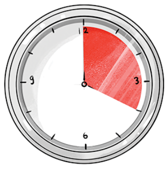

Laisser reposer

NB : Le filtrage avec un tissu ou éponge n'enlève pas les microbes !
Il peut néanmoins enlever un peu de saleté pour pouvoir la désinfecter avec l'eau de javel après

08 SESSION 8 : LA MALNUTRITION

8.1. DEFINITION :

La Malnutrition désigne un état pathologique causé par la déficience[1] d'un ou plusieurs nutriments essentiels à la croissance normale et au bon fonctionnement de l'organisme.

Il existe deux principales causes immédiates de la malnutrition :

- Apports alimentaires inadéquats : Faible pratique de l'allaitement exclusif, arrêt brusque et précoce de l'allaitement, interdits alimentaires, repas non équilibrés et/ou insuffisants)
- Maladies responsables d'une augmentation des besoins non couverts et /ou un manque d'appétit (Rougeole, diarrhée chronique, paludisme répété, SIDA, parasitoses intestinales etc.).

Les Conséquences de la malnutrition sur le développement de l'enfant

- La malnutrition affaiblit l'organisme de l'enfant
- Elle favorise le développement des autres maladies
- L'enfant malnutri a plus de risque de mourir .

Lorsqu'une maman vient avec son enfant pour consulter pour la toux, la diarrhée ou la fièvre ; il faut systématiquement vérifier l'état nutritionnel par la recherche des œdèmes et la mesure du PB>

Le groupe-cible pour la mesure du PB est constitué par les enfants de 6 à 59 mois. En-dessous de 6 mois la mesure du périmètre brachial (PB) n'est pas fiable et ne détectera pas la malnutrition.

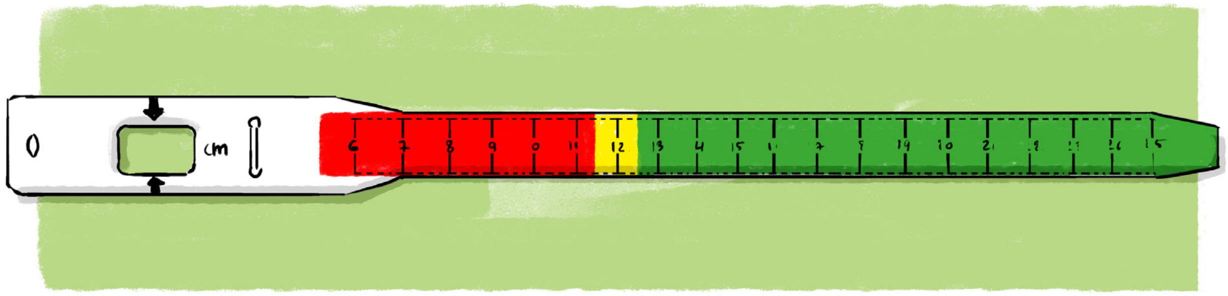

6.2. IDENTIFICATION DE L'ENFANT MALNUTRI

COMMENT MESURER LE PERIMETRE BRACHIAL

Comment prendre le tour du bras à mi-hauteur

1. Préparer le matériel à portée de main (MUAC)
2. Expliquer au malade et au parent ce que l'on va faire.
3. Demander à la mère de repousser les manches de l'habit de l'enfant

[1] Ne parler que de la malnutrition carentielle pour éviter de semer la confusion chez les participants. Dans la mesure où c'est la seule partie abordee dans la PCIMA.

Ruban pour la mesure du Périmètre brachial

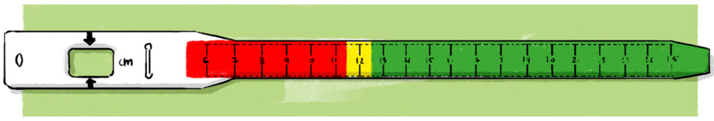

Repérer l'extrémité le sommet de l'épaule
Identifier l'extrémité de l'épaule
Identifier l'extrémité du coude

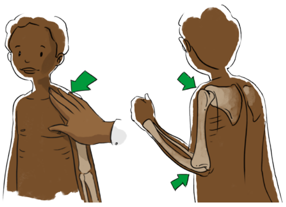

Placer le ruban à l'extrémité de l'épaule
Dérouler le ruban jusqu'à l'extrémité du coude
Noter le point médian

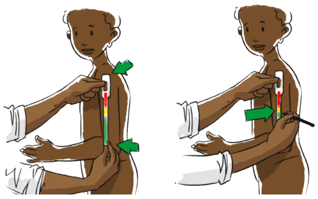

Maintenir le ruban
pas trop lâche
pas trop serré

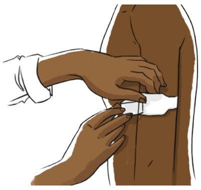

Exemple de ruban
trop serré

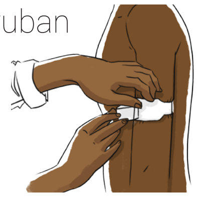

Exemple de ruban
trop lâche

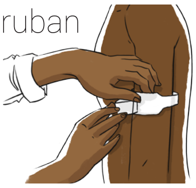

Faire la lecure (Position correcte pour mesurer le périmètre brachial)
Noter le PB dans le carnet et dans le registre

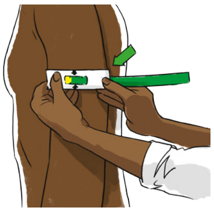

Comment prendre le Périmètre Brachial (PB) à un enfant

Fiche d'apprentissage

TÂCHES / GESTES	NOMBRE DE PRATIQUES				
	1	2	3	4	5
1. Préparer le matériel à portée de main (MUAC)					
2. Expliquer au malade ou au parent ce que l'on va faire.					
3. Demander à la mère de repousser les manches de l'habit de l'enfant					
Repérage du point médian					
4. Repérer l'extrémité le sommet de l'épaule					
5. Identifier l'extrémité de l'épaule					
6. Identifier l'extrémité du coude					
7. Placer le ruban à l'extrémité de l'épaule					
8. Dérouler le ruban jusqu'à l'extrémité du coude					
9. Noter le point médian (le milieu)					
Mesure proprement dite du PB					
10. Entourer le ruban pas trop lâche et pas trop serré autour du bras de l'enfant au point médian					
11. Faire la lecture					
12. Noter le PB sur la fiche de prise en charge et dans le registre					
TOTAL					

COMMENT RECHERCHER LES OEDEMES

Les enfants avec des œdèmes nutritionnels sont directement identifiés comme étant malnutris aiguë sévère. Ces enfants sont à haut risque de mortalité et doivent être rapidement traités dans un programme de prise en charge de la malnutrition aiguë.

Les œdèmes sont évalués comme suit :

- On exerce une pression normale avec le pouce sur les deux pieds pendant au moins trois secondes.
- Si l'empreinte du pouce persiste sur les deux pieds, alors l'enfant présente des œdèmes nutritionnels.
- **Seuls les enfants avec des œdèmes bilatéraux sont enregistrés comme ayant des œdèmes nutritionnels.**

NB : Vous devez tester avec la pression de votre doigt ! Il ne suffit pas uniquement de regarder

Les œdèmes de la malnutrition sont toujours bilatéraux, mous, indolores et gardent la trace.

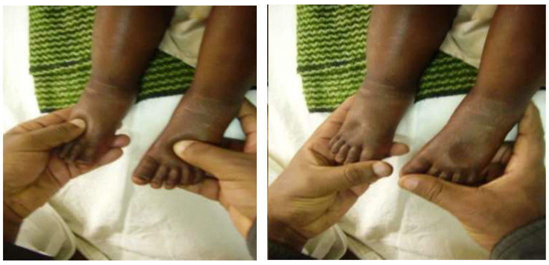

SEVERITE DES OEDEMES	CODIFICATION
Oedèmes légers : des 2 pieds	+
Oedèmes modéres : des 2 pieds et de la partie inférieure des 2 jambes, ou les 2 mains et la partie inférieure des 2 avant-bras.<br>Intermédiaire entre le degrè d'oedèmes légers et sévères	++
Oedèmes sévères : géneralisé soit incluant les 2 pieds, jambes, mains, bras et le visage	+++

Une fois les signes de danger recherchés et s'il n'y a pas d'œdème des pieds, la malnutrition aiguë est recherche chez tous les enfants âgés de 6 à 59 mois par la mesure du PB.

L'ASC reporte sur la fiche la mesure du PB et classe ainsi l'enfant dans une des catégories : MAM (malnutrition aiguë modérée) si le PB est compris entre 125 mm et 115 mm (partie jaune), MAS (malnutrition aiguë sévère) si le PB est inférieur à 115 mm (partie rouge) ou pas de malnutrition si le PB est supérieur ou égale à 125 mm (partie verte).

SIGNES	CLASSIFICATION
PB inférieur à 115 mm (rouge) et/ou Œdèmes aux 2 pieds 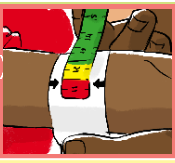 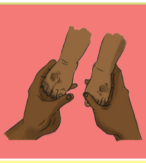	MALNUTRITION AIGUE SEVERE
PB compris entre 115 mm et 125 mm (jaune) 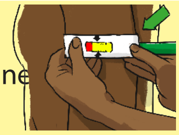	MALNUTRITION AIGUE MODEREE
PB dans la bande verte et pas d'œdèmes des pieds 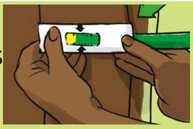	PAS DE MALNUTRITION

L'enfant atteint de MAS est référé au niveau supérieur, les enfants atteints de MAM sont orientés vers le CRENAM et hebdomadairement suivi pour détecter et référer la MAS dès sa survenue.

09 SESSION 9 : LA VACCINATION

Quelles sont les maladies qu'on peut prévenir par la vaccination en Mauritanie ?

La vaccination protège contre :

1. La tuberculose,
2. La poliomyélite,
3. La diphtérie,
4. La coqueluche,
5. Le tétanos,
6. L'hépatite B,
7. Les infections dues à l'Haemophilus influenza de type B (Hib),
8. La rougeole
9. Les infections à Pneumocoque
10. Les infections gastro-entérites à rota virus

Quel est le rôle de l'ASC dans la vaccination ?

L'ASC doit expliquer aux mères :

- L'intérêt de la vaccination
- La nécessité de garder soigneusement les cartes de vaccination qui permettent de suivre l'état vaccinal des enfants et des femmes en âge de procréer. Il doit vérifier que tout le village est averti ainsi que les campements environnants.

S'il y a un poste de santé proche du village doté d'une unité de vaccination, l'ASC doit s'assurer que les mères y amènent les enfants pour la vaccination devant avoir lieu et note la date de la future vaccination pour veiller avec les mères le respect des rendez-vous jusqu'à immunisation complète des enfants.

Le même intérêt doit être manifesté pour la vaccination des femmes enceintes et le suivi de leur grossesse comme leur état nutritionnel.

Aider l'équipe lors des séances de vaccination en stratégies mobiles et avancées en recherchant activement les enfants cibles non vaccinés.

Quelle est la population cible de vaccination ?

- Tous les enfants de 0 à 11 mois pour le programme régulier
- Toutes les femmes enceintes (protection contre le tétanos et la diphtérie)

Quel est le calendrier de la vaccination ?

Les enfants sont vaccinés selon le calendrier suivant :

ANTIGÈNES	AGE RECOMMANDÉ
B CG	A la Naissance
Hépatite B	A la Naissance (les 1ères 24h)
Polio 0	A la naissance
Polio 1 + Penta1 + Pneumo1 + Rota1	6 semaines de la naissance
Polio 2 + Penta2 + Pneumo2 + Rota2	10 semaines de la naissance
Polio 3 + Penta3 + Pneumo3 + VPI	14 semaines de la naissance
VAR et VAA	9 mois

Les femmes en âge de procréer de 14 à 44 ans Vaccin contre le Tétanos et la diphtérie

DOSE D'ANTIGÈNES	CALENDRIER ET L'INTERVALLE MINIMAL	DURÉE DE PROTECTION
Td 1	Au 1er contact	Aucune
Td 2	1 mois (28 j) après Td1	3 ans
Td 3	6 mois après Td2	5 ans
Td 4	1 an après Td3	10 ans
Td 5	1 an après Td4	à vie

MANIFESTATIONS ADVERSE POSTE INJECTIONS (MAPI)

- Incident médical inquiétant qui se produit à la suite d'une vaccination et dont on pense qu'il est dû à celle-ci.

  Réactions locales (une réaction douloureuse et sensible au point d'injection qui disparaît dans les 24 heures suivant la vaccination) ; Fièvre modérée ne dépassant pas 39° qui dure 24- 48 heures

Ces MAPI mineures doivent être signalés automatiquement aux structures de santé

Type de MAPI

1. MAPI Mineures :

   Réaction légère qui n'entraine pas une hospitalisation ou un décès

2. MAPI GRAVE (majeures) :

   Réaction qui entraîne un décès ou une hospitalisation

Causes probable

- Erreurs imputables au programme :
  - Mauvaise conservation au cours du transport, du stockage.
  - Problèmes de manipulation, reconstitution, de l'administration, mauvais diluants…
- Réactions liées au vaccin :
  Évènements médicaux dus aux propriétés du vaccin/ réaction de l'hôte à l'antigène, anxiété…
- Coïncidence
  Évènement médical qui aurait pu survenir indépendamment de la vaccination

10 SESSION 10 : SURVEILLANCE A BASE COMMUNAUTAIRE

La surveillance communautaire c'est la méthode de la surveillance des maladies au niveau communautaire.

10.1. LES ACTEURS DE LA SURVEILLANCE COMMUNAUTAIRE :

- La communauté :

  Rapporte rapidement les rumeurs sur les maladies ou tout autre événement inhabituel au niveau des relais communautaires et des agents de santé.

- Le relais communautaire :
  - Organiser des séances de sensibilisation sur les maladies sous surveillance
  - Organiser des visites à domicile ;
  - Informer l'ASC de toute rumeur sur les maladies ou tout autre événement inhabituel de santé dans la communauté
- Les agents de santé responsables des USB :
  - Rechercher activement les cas suspects des maladies ou tout autre événement inhabituel dans la communauté ;
  - Rapporter rapidement aux PS les cas des maladies suspectes dans la communauté ;
  - Accompagner physiquement ou par les conseils les familles et/ou les malades aux PS
  - Sensibiliser la communauté sur les maladies sous surveillance ;
  - Participer aux réunions, aux formations et aux activités de planification ;
  - Participer aux enquêtes et aux activités de riposte sur la surveillance dans la communauté ;
  - Assurer la retro information du poste de santé vers la communauté ;
  - Organiser les activités sur terrain ;
  - Organiser des visites à domicile ;
  - Enregistrer les naissances et les décès

10.2. LES OUTILS DE LA SURVEILLANCE A BASE COMMUNAUTAIRE

Les principaux outils de la surveillance à base communautaire sont :

- Les définitions de cas
- les fiches de rapportage de cas.

10.3. DEFINTION DES MALADIES :

Dans le cadre de la surveillance communautaire, une priorité est accordée aux maladies transmissibles parmi lesquelles celles évitables par la vaccination dont :

La PFA/Polio, la Rougeole, le Tétanos maternel et néonatal, la Méningite, la fièvre jaune, les Fièvres hémorragiques virales et le Choléra.

L'accent est mis dans un premier temps sur ces maladies parce qu'elles sont les plus fréquentes, de diagnostic facile, et pour lesquelles les moyens de prévention et de riposte sont disponibles.

Il y a lieu de souligner qu'en plus des maladies citées, on peut associer d'une façon ponctuelle, les membres de la communauté à la surveillance de toute autre maladie ou événement sanitaire inhabituel.

NUMEROS	DEFINITION DES CAS SIMPLIFIES POUR LA SURVEILLANCE DES MALADIES	
	MALADIE / AFFECTION / EVÉNEMENT	DÉFINITION DE CAS STANDARDISÉE
1	LA POLIOMYELITE / La PFA (paralysie flasque aiguë)	Tout enfant de moins de 15 ans qui rampait ou marchait et brusquement ne rampe plus ou ne marche plus
2	LA ROUGEOLE	Toute personne, notamment les jeunes enfants, manifestant la fièvre et une Éruption cutanée
3	LE TETANOS NEO NATALE	Tout nouveau-né capable de pleurer et de prendre le sein normalement pendant les 2 premiers jours de sa vie, et qui, entre le 3ème et 28ème jour, ne peut plus téter normalement, devient raide et/ou a des convulsions
4	TETANOS MATERNEL /DECES MATERNELS	Toute femme présentant des contractions musculaires localisées ou généralisées ou qui en est décédée dans les 6 semaines qui suivent l'accouchement.
5	LA MENINGITE	Tout individu souffrant de la fièvre et de raideur du cou.
6	LA FIEVRE JAUNE	Tout individu souffrant de la fièvre et du jaunissement des yeux ou de la peau (plantes des pieds, ongles, paumes des mains).
7	LE CHOLERA	Tout individu âgé de 5 ans et plus ayant des selles liquides (eau de riz) abondantes
8	LES FIEVRES HEMORRAGIQUES VIRALES	Tout individu atteint de fièvre et présentant des saignements ou qui en est décédé.
